# Importance of EMT Factor ZEB1 in cDC1 “MutuDC Line” Mediated Induction of Th1 Immune Response

**DOI:** 10.3389/fimmu.2018.02604

**Published:** 2018-11-13

**Authors:** Shuchi Smita, Abdul Ahad, Arup Ghosh, Viplov K. Biswas, Marianna M. Koga, Bhawna Gupta, Hans Acha-Orbea, Sunil K. Raghav

**Affiliations:** ^1^Immuno-genomics and Systems Biology Laboratory, Institute of Life Sciences (ILS), Bhubaneswar, India; ^2^Manipal Academy of Higher Education, Manipal, India; ^3^Department of Biotechnology, Kalinga Institute of Industrial Technology (KIIT), Bhubaneswar, India; ^4^Department of Biochemistry CIIL, University of Lausanne (UNIL), Epalinges, Switzerland

**Keywords:** ZEB1, cDC1 dendritic cells, integrative genomics, Th2 response, ChIP-seq, RNA-seq, helminth infection, immune modulation

## Abstract

The role of Epithelial to Mesenchymal Transition (EMT) factor Zeb1 is well defined in metastasis and cancer progression but it's importance in dendritic cells (DCs) is unexplored until now. For the first time we report here that Zeb1 controls immunogenic responses of CD8α^+^ conventional Type-I (cDC1) DCs. We found that ZEB1 expression increases significantly after TLR9 stimulation and its depletion impairs activation, co-stimulation and secretion of important cytokines like IL-6, IL-10 and IL-12 in cDC1 MutuDC line. We further confirmed our findings in primary cDC1 DCs derived from bone marrow. Co-culture of these Zeb1 knock down (KD) DCs with OT-II CD4^+^ T helper cells skewed their differentiation toward Th2 subtype. Moreover, adoptive transfer of activated Zeb1 KD DCs cleared intestinal worms in helminth infected mice by increasing Th2 responses *in vivo*. Integrative genomic analysis showed Zeb1 as an activator of immune response genes in cDC1 MutuDCs as compared to other pathway genes. In addition, differentially regulated genes in Zeb1 KD RNA-seq showed significant enrichment of Th2 activation pathways supporting our *in vitro* findings. Mechanistically, we showed that decreased IL-12 secreted by Zeb1 KD DCs is the plausible mechanism for increased Th2 differentiation. Collectively our data demonstrate that Zeb1 could be targeted in DCs to modulate T-cell mediated adaptive immune responses.

## Introduction

Dendritic cells (DCs) are potent antigen presenting cells that play a pivotal role in developing immune responses as they govern both the initiation and polarization of adaptive immunity (1–5). The complex classification and nomenclature of DCs has now been refined into two levels, conventional or classical DCs comprising cDC1 (CD8α^+^ and CD103^+^) and cDC2 (CD11b^+^ and CD172a^+^ DCs) depending on their distinct developmental pathways, and the plasmacytoid DCs (pDCs) (6–8). Genetic and functional studies have revealed that CD8α^+^ (Lymphoid-resident DCs) and CD103^+^ (Non-lymphoid tissue resident migratory DCs) DCs are specialized in antigen cross-presentation and polarization of Th cells into Th1 subset in response to stimulation via Toll Like receptor (TLR) ligands such as CpG for TLR9 and poly-IC for TLR3 (6, 7, 9–12). Upon pathogen encounter, DCs are activated leading to their maturation and migration toward secondary lymph nodes where they instruct T helper (Th) cell differentiation into different subtypes in a signal dependent manner (13–19). Among the many Th cell subsets, Th1 cells are critical for host defense against intracellular pathogens, while Th2 cells defend extracellular parasites (12, 15–17). Priming of Th cells toward Th1 requires inflammatory cytokine IL-12 whereas Th17 subtype depends on IL-6 and IL-23 cytokines produced by DCs (20, 21). In contrast, it has been widely accepted that DCs do not produce the Th2 speciation cytokines like IL-4 and IL-13. Therefore, decreased secretion of Th1 or Th17 promoting cytokines by DCs could induce the Th2 cell differentiation as a default outcome (22–24). Besides, considerable evidences suggest that DCs are required for optimal Th2 cell priming *in vivo* and expression of co-stimulatory molecules like OX40L or the Notch ligand Jagged-1 by DCs promotes Th2 cell priming (25, 26). On the other hand, it is explicitly known that cDC1 are prone to induce Th1 responses whereas cDC2 cells provide cooperative signal for Th2 responses where the IL-4 cytokine remains the key-determining factor for their polarization (27–29). Interestingly, there are several reports showing upregulation of Th2 transcription factor GATA3 through IL-4 by activating STAT5 and STAT6 transcription factors (TFs), but few of them indicate that GATA3 expression can be independent of IL-4 as well (28, 30). Apart from signaling molecules, it has been reported that IRF4 depleted DCs are unable to induce Th2 differentiation (28, 31, 32), whereas increased KLF2 in DCs negatively regulates Th2 induction (33).

E-Box motif binding TF Zeb1 is a member of Zinc finger TF family, a known EMT master regulator. TGFβ signaling is one of the main mechanisms promoting EMT and is known to induce Zeb1 through SMAD signaling which in turn is well documented to repress E-cadherin (Cdh1) expression in epithelial cells (34, 35). The mir200 family members are predominantly present in epithelial cells and fine-tune the transcript expression of Zeb1 through feedback regulation (34, 36). In breast cancer cells, knock down of Zeb1 inhibits pro-inflammatory cytokines including IL-6 and IL-8 (37). Similarly, it has been widely reported that EMT in tumors is positively induced by inflammation (36, 38–41). In contrast, Zeb1 has been reported to repress IL-2 by recruiting CTBP2 at its proximal promoter in T-cells irrespective of activation (42). There are reports suggesting higher expression of Zeb1 in migratory Langerhans cells, pertinent for their migration to secondary lymph nodes to present antigens to Th cells (43). This indicated that Zeb1 might be playing an important role in cDC1 axis of immune biology beyond just migratory properties. A forward genetic screen also revealed Zeb1 requirement for marginal zone of peritoneal B-1 B-cell development, T-cell development, germinal center formation, and memory B-cell responses (44). Though Zeb1 has been widely studied in cancer biology, few evidences with immunity and inflammation make it a potential candidate to look upon for its role in cDCs trajectory.

Here in this study, we investigated the role of Zeb1 in CD8α^+^ cDC1 DCs and found it to be pertinent for their activation, co-stimulation and secretion of important immune response cytokines like IL-10 and IL-12. As a result, Zeb1 depleted DCs generated a strong Th2 phenotype *ex vivo* and *in vivo*, independent of IL-4 cytokine. Integrative genomic analysis demonstrated that Zeb1 has an indirect control on important cDC1 response cytokines and it does not act as a global repressor of immune response genes in DCs.

## Methods

### Dendritic cell (DC) culture

Here in this study we have used CD8α^+^ cDC1 MutuDC line recently developed by Prof. Hans Acha-Orbea's group. They have extensively characterized and compared these DC lines with primary CD8α^+^ cDC1 DCs and reported that they perfectly mimic *ex vivo* immature CD8α^+^ DCs isolated from spleen of C57BL/6 mice (9). The DCs were grown in IMDM-glutamax (GIBCO) buffered with NaHCO_3_ and supplemented with 8–10% heat inactivated FCS (tested for endotoxin toxicity toward DC cultures), 10 mM HEPES (GIBCO 15630), 50 μM β-Mercaptoethanol (GIBCO 31350), and 50 U/mL of penicillin and 50 μg/mL streptomycin (GIBCO 15070). The cells were maintained at 37°C in a humidified incubator with 5% CO_2_. These DCs were dissociated with short incubation in non-enzymatic, 5 mM EDTA-based cell dissociation buffer (5 mM EDTA in 20 mM HEPES-PBS) at 37°C.

For *in vitro* experiments, the DCs were plated in 6-well plates at a density of 5 × 10^5^ cells/ml overnight. The cells were then challenged with different activation media containing TLR9 agonist CpG-B (Invivogen, cat no. tlrl-1826), TLR3 agonist pIC (Invivogen, cat no. tlrl-pic) and CpG+pIC for 2, 6, and 12 h. For performing RT-qPCR analysis the cells were washed in the plate once with PBS followed by addition of RNA-later (LBP) lysis buffer (Macherey-Nagel) for lysis of cells. The plates were then stored at −80°C until further RNA isolation and processing of samples.

### Generation of stable Zeb1 KD CD8α^+^ MutuDCs

For generating stable Zeb1 knockdown and corresponding control DCs, lentiviral vector pLKO.1 (Sigma) containing three different Zeb1-specific shRNAs or control shRNA were used. Viral particles packaged with shRNA expressing transfer plasmids were produced in 293T cells using Cal-Phos (CaPO_4_) mammalian transfection kit (Clontech) according to an optimized protocol (45). 293T cells were transfected with transfer plasmids containing three different Zeb1 shRNAs or control shRNAs along with packaging plasmids (pCMVR8.74 and pMD2G). After 12–14 h the culture medium was replenished and supernatant containing viral particles were collected after 24 h in 50 ml conical tubes. Viral particle-containing culture supernatant was filtered through 0.45 μm syringe filters (PES filters) and preserved at −80°C in small aliquots. For transduction of shRNA containing viruses in CD8α^+^ cDC1 MutuDC lines, the cells were plated at a density of 1.5 × 10^5^ cells/well of 12 well plate followed by transduction with virus particles containing supernatant. The media was replaced with fresh media after 12 h of virus incubation with DCs followed by addition of 1 μg/ml puromycin selection medium after 72 h of media replacement. The cells were puromycin selected for 2–3 weeks to get stable Zeb1 KD cells. The cells were also transduced with control shRNA-containing viruses to develop control cells for analysis comparisons. Efficiency of Zeb1 KD was quantified using Zeb1 gene specific primers by RT-qPCR (Supplementary Table 8). The shRNA that showed significant and maximum decrease in Zeb1 gene transcript levels compared to control transduced cells were used for further detailed study.

### RNA isolation and RT-qPCR

The cells preserved in LBP lysis buffer for RT-qPCR experiments were first taken out from −80°C and thawed by placing the plates/tubes on ice. Total RNA was isolated using NucleoSpin RNA Plus kit (Machery-Nagel) according to manufacturer's protocol. RNA concentration was estimated by nanodrop (Thermo) and then 1 μg of total RNA was used to prepare cDNA using High capacity cDNA Reverse Transcriptase kit (Applied biosystems). Quantiative PCR was performed using SYBR Green master mix (Roche) and PCR amplification was monitored in real-time using LightCycler-480 Instrument (Roche). Primer oligonucleotides for qPCR were designed using universal probe library assay design system (Roche) and the primer pairs used are listed in Supplementary Table 8. Primers were optimized for linear and single product amplification by performing standard curve assays.

### Flow cytometry (FACS)

Flow cytometric analyses of *in vitro* and *ex vivo* cultured cells were performed using well established protocol for FACS staining and analysis. For surface and intracellular (IC) staining 5^*^10^5^ and 1.5^*^10^6^ cells were seeded respectively and stimulated with CpG, pIC and CpG + pIC for 12 h. After dissociation from plates, the cultured cells were washed with FACS buffer (3% FCS in PBS, 5 mM EDTA) followed by re-suspension in surface staining buffer. After washing, fluorochrome conjugated antibodies for proteins of interest were added to the cells as a cocktail (Supplementary Table 8). For intracellular (IC) staining of cytokines the cells were first fixed with 2% paraformaldehyde followed by permeabilisation using 1x permeabilisation buffer (eBiosciences). The fixed cells were then resuspended in intracellular staining buffer and stained with fluorochrome tagged antibodies for selected cytokines. For optimal staining the cells were incubated with antibodies for 30 min in dark at 4°C. After incubation the cells were washed twice with FACS wash buffer and then acquired for differential expression analysis using LSRII fortessa flow cytometer (BD Biosciences). The acquired data was analyzed using FlowJo-X software (Treestar).

We used single color antibody stained cells as controls using pooled cells from all untreated and treated conditions for compensation and gating of positive population. In one of the biological replicates for immune profiling experiments, we used Florescence Minus One controls (FMO) to gate cells and for compensation. We found that single color stained and unstained negative control cells were giving similar results and therefore we didn't include FMOs in all our further experiments. From live cell population (high GFP positive cells) first we removed doublet cell population and then similar gates were employed for both control and Zeb1 KD DCs to observe any percentage cell population differences in surface markers and intracellular cytokines. Unstimulated DCs do not secrete cytokines therefore we used these cells stained with similar cocktail of antibodies for gating the cytokine positive cell population. In addition, we also analyzed for Median Florescence Intensity (MFI) shifts for each population in replicates to observe overall activation/co-stimulation markers and cytokines in control and Zeb1 depleted DCs.

### Bio-plex assay for cytokine quantitation from cell culture supernatants

Bio-Plex assay (multiplex ELISA) was used to estimate the cytokine levels secreted in the cell culture supernatants of Zeb1 KD and control DCs after 12 h of CpG stimulation. After culture, the supernatants were stored at −80°C in small aliquots until analysis. Cytokine levels were estimated using 23-plex-mouse cytokine assay kit following the vendor recommended protocol (Biorad).

### Generation of bone marrow derived DCs (BMDCs) for *ex-vivo* studies

Six to eight-week-old female C57BL/6 mice were killed by cervical dislocation and disinfected using 75% ethanol. The tibias and femurs were removed under sterile conditions, then soaked in RPMI-1640 medium supplemented with 10% FBS. Both ends of the bone were cut off with scissors, and the needle of a 1-mL syringe was inserted into the bone cavity to rinse the bone marrow out of the cavity into a sterile culture dish with RPMI-1640 medium (46). The cell suspension in the dish was collected and centrifuged at 350 g for 5 min, and the supernatant was discarded. The cell pellet was suspended with 1X RBC lysis buffer (Tonbo: TNB-4300) to lyse the RBCs and incubated for 5–10 min on ice. Cell clumps were then passed through a 70 μm strainer to obtain single cell suspensions. The lysed cells were washed once with RPMI-1640, counted and used for differentiation into DCs.

We followed a well-established protocol for differentiation of BMDCs with slight modifications (47). The cells, suspended in RPMI-1640 medium supplemented with 10% FBS, were distributed into 6-well plates at a density of 1 × 10^6^ cell/ml/well. Subsequently, 1μl/ml of FLT3L containing sera (derived from Flt3L expressing mice, gift from Hans Acha-Orbea, UNIL, Lausanne, Switzerland) was added into the medium. The cells were cultured at 37°C in an incubator containing 5% CO_2_ and left untouched for 5 days. On day 5, the suspended and loosely attached cells were collected, washed and counted. The cells were plated into 24-well plate for lentiviral transduction using concentrated viruses at a density of 0.4^*^10^6^ cells/well for each Zeb1 shRNA and Control shRNA. After 72 h the cells were stimulated with CpG for 12 h and then immune-profiling was done at protein level using flow cytometry for observed markers that were found to be differentially regulated in CD8α^+^ cDC1 cells *in vitro*. The antibodies used for staining were same as used for *in vitro* experiments.

### Co-culture of DCs with CD4^+^ T cells for assessing T-cell proliferation and differentiation

DC-T cell co-culture experiments were performed as described before (9). Naïve CD4^+^ T cells were purified from spleen of TCR-transgenic OT-II mice using CD4^+^ T cell isolation kit (EasySepTM Mouse CD4^+^T cells isolation Kit, Stem Cell Technologies). Zeb1 KD and control CD8α^+^ cDC1 DCs were seeded at a density of 10,000 cells/well in round bottom 96 well plates followed by pulsing with OVA peptide (aa 323-339) and CpG for 2 h. After 2 h, purified OT-II T cells were added at the density of 100,000 cells/well (1:10 ratio) (48). Then T-cell proliferation and differentiation into distinct Th subtypes Th1, Th2, Th17 and Tregs were analyzed by FACS. Proliferation was measured using an amine based dye (eFluor 670). The rate of T-cell proliferation was inversely proportional to the Median Fluorescence Intensity (MFI) measured in FACS after 72 h of co-culture. For Th cell differentiation profiling after 96 h, the co-cultured T cells were re-stimulated with PMA (10 ng/mL) and Ionomycin (500 ng/mL) and followed by Brefeldin-A (10 μg/mL) treatment for 5 h to block the intracellular cytokines from being secreted. After 5 h, fluorochrome conjugated antibodies specific to different T cell subtypes were used to profile T cells into Th1 (Tbet and IFNγ), Th2 (GATA3, IL-13), Tregs (CD25, FoxP3) and Th17 (IL-17) (49). For gating effector T cells we used CD44 as a marker (see Supplementary Table 8 for details of antibodies).

To confirm the default Th2 program recombinant IL-12 (5 ng/ml) and anti-IL4 (5 μg/ml) was used in OT-II co-culture experiments to confirm any perturbation in Th subtype differentiation using similar antibodies for various subtypes.

### Chromatin immuno-precipitation (ChIP) for Zeb1

The ChIP for Zeb1 was performed according to the methods optimized previously by Raghav and Meyer's lab (50, 51). For ChIP assays, 30^*^10^6^ CD8a^+^ cDC1 MutuDCs were seeded in 15 cm^2^ plates and prepared for ChIP by 10 min cross-linking with 1% formaldehyde (sigma) at room temperature followed by quenching using 2.5 M glycine (sigma) for 10 min. The plates were placed on ice and the cells were scraped and collected in 50 ml conical tubes. The cells were then washed three times using cold 1x PBS at 2,000 rpm for 10 min at 4°C and the cell pellets were stored at −80°C. At the day of the ChIP experiment, the cells were thawed on ice followed by lysis using nuclei extraction buffer (50 mM HEPES-NaOH pH 7.5, 140 mM NaCl, 1 mM EDTA pH 8.0, 10% glycerol, 0.5% NP-40, 0.25% Triton-X100) supplemented with protease and phosphatase inhibitors (Roche) for 10 min at 4°C on rocker shaker. The prepared nuclei were then washed using protein extraction buffer (200 mMNaCl, 1 mM EDTA pH 8.0, 0.5 mM EGTA pH 8.0, 10 mM Tris-Cl pH 8.0) supplemented with a protease and phosphatase inhibitors (Roche) at room temperature for 10 min. Washed nuclei were resuspended in chromatin extraction buffer (1 mM EDTA pH 8.0, 0.5 mM EGTA pH 8.0, 10 mM Tris-Cl pH 8.0 and 1% TritonX-100) supplemented with protease and phosphatase inhibitors (Roche) and incubated for 20 min on ice for equilibration. The chromatin was fragmented using a Bioruptor (Diagenode) sonicator for 30 min using high amplitude and 30s ON & 30s OFF cycles to obtain 200-500 bp size fragments. A cooling unit was used to circulate the cold water during sonication to avoid de-crosslinking because of overheating. After sonication, chromatin length was checked in agarose gel. The fragmented chromatin was centrifuged at 10,000 rpm for 5 min and then clear supernatant was collected in 15 ml conical tubes. The DNA concentration of the chromatin was estimated using a Nano-Drop (Thermo) and the chromatin was diluted with ChIP dilution buffer (1 mM EDTA pH 8.0, 10 mM Tris-Cl pH 8.0 and 1% TritonX-100 containing protease and phosphatase inhibitors) to use 150 μg/ml of chromatin for each IP. BSA and ssDNA (Salmon Sperm DNA) preblocked protein-A sepharose (80 μl/IP) beads were added to the samples on ice and incubated for 2 h to remove non-specific-binding chromatin. To the supernatant, 25 μl of rabbit polyclonal anti-Zeb1 (Santa Cruz H-102) were added to immunoprecipitate the chromatin complex at 4°C overnight on rocker shaker. After the overnight incubation, 50 μl blocked beads were added to each sample and incubated for 2.5 h at 4°C to pull down the respective antibody-chromatin complexes. The beads were then washed three times with low salt wash buffer (20 mM Tris-Cl pH 8.0, 150 mM NaCl, 2 mM EDTA pH 8.0, 0.1% SDS, 1% TritonX-100) followed by two washes with high salt wash buffer (20 mM Tris-Cl pH 8.0, 500 mM NaCl, 2 mM EDTA pH 8.0, 0.1% SDS, 1% TritonX- 100), lithium chloride wash buffer (10 mM Tris-Cl pH 8.0, 0.25 M LiCl, 1 mM EDTA pH 8.0, 1% NP-40, 1% sodium deoxycholate) and Tris-EDTA (TE) buffer (10 mM Tris-Cl pH 8.0, 1 mM EDTA pH 8.0). After removing the wash buffer completely, protein-bound chromatin complexes were eluted from beads for 30 min using elution buffer (100 mM NaHCO_3_ and 1% SDS in milli-Q water). The eluted chromatin was then reverse crosslinked by incubating the eluted supernatant at 65°C overnight on a heat block after adding 8 μl of 5 M NaCl. Next day DNA was purified from the reverse cross-linked chromatin by proteinase-K and RNase digestion followed by purification using PCR purification kit (Qiagen). The purified DNA was eluted in 40 μl of elution buffer.

### ChIP-/RNA-seq library preparation for next generation sequencing (NGS)

The RNA-seq library preparation was performed for Zeb1 KD and control cDC1 MutuDCs at 0, 6, and 12 h after CpG activation. As we did time kinetics we included two independent biological replicates to identify the Zeb1 depletion mediated global transcriptome changes. For RNA-seq library preparation 2 μg of total RNA was used to isolate mRNA through magnetic beads using mRNA isolation kit (PolyA mRNA isolation Module, NEB) followed by RNA-seq library preparation using mRNA library preparation kit (NEB) strictly following the vendor recommended protocol. After library preparation concentration of libraries were estimated using qubit 2.0 (Invitrogen) and the recommended fragmentation sizes were confirmed by Bio-analyzer (Agilent). For ChIP-seq library preparation, 30 μl of ChIP-DNA was processed for library preparation according to ChIP-seq library preparation recommended protocol (NEB). After library preparation and quality check using Bio-analyzer, the libraries were send to NGS service provider for Illumina sequencing using Hiseq-2500 instrument.

### Western blotting

Cells were collected in RIPA buffer (0.5 M EDTA, 1 M Tris-Cl pH7.5, 1 M NaCl, 200 mM PMSF, 10% NP-40, 10% SDS, 5% sodium deoxycholate, 1 M sodium orthovanadate and 1X Roche protease inhibitor) before and after CpG stimulation at different time points (0, 1, 2, 6, & 12 h). Cells were lysed completely by sonicating the samples in Bioruptor (Diagenode) for 10 min using high amplitude and 30s ON & 30s OFF cycles. Protein concentrations were measured in 96 well plate using BCA protein assay kit (BioRad).

### Adoptive transfer of DCs in helminth (*Heligmosomoides polygyrus)* infection mice model

For DC Adoptive transfer experiments we took 6–8 week old female C57BL/6 mice and infected them with 200 infective L3 larvae/mice in PBS though oral gavage. Prof. Nicola Harris from EPFL (École Polytechnique Fédérale de Lausanne), Lausanne, Switzerland, provided the infective L3 larvae. The larvae were hatched from fecal charcoal cultures at day 7 after collection (52). After 7 days of infection, mice were treated with 100 μl of anti-CD8b antibody/mice followed by adoptive transfer of 10^*^10^6^ CpG pulsed Zeb1 KD and control CD8α^+^ cDC1 MutuDCs in sterile PBS intra-peritoneally (IP). Two booster doses of 5^*^10^6^ cells pulsed with CpG were adoptively transferred consequtively after 48 h. After adoptive transfer of DCs the feces from infected animals were collected for worm eggs counting after every 24 h time period till day 31 using a well-optimized protocol. After observing a significant difference in egg count between Zeb1 KD DC treated and control animals, four mice from each group were sacrificed for detailed T cell profiling from mesenteric lymph nodes and the helminth worm counting from the intestine of the dissected animals. The intestines were longitudinally opened and flipped to count the worms and to take pictures. The T cell differentiation into Th1, Th2, Tregs, and Th17 subtypes was assessed using FACS as detailed above. This mouse experiment was performed following the institutional animal ethics guidelines after taking due approval from the institutional animal ethics committee at ILS, Bhubaneswar, India.

### Mixed lymphocyte reaction (MLR)

MLR setup was performed following well-documented protocol with slight modifications (49). The Zeb1 KD and control DCs were co-cultured with allogenic T cells from spleen of Balb/C mice. The Zeb1 KD and control CD8α^+^ DCs were seeded at a density of 20,000 cells/well in a round bottom 96 well plates followed by challenge with CpG for 2 h. After 2 h, splenocytes containing mostly T cells from 6–8 week old Balb/C mice were added at the density of 200,000 cells/well (1:10 ratio). After 4 days (96 h) of co-culture, T-cell differentiation was assessed using same method as for OT-II co-culture.

## NGS analysis

### Quality control & preprocessing

The quality of RNA-seq and ChIP-seq reads from all sequencing experiments were determined using FastQC v0.11.5 (https://www.bioinformatics.babraham.ac.uk/projects/fastqc/) tool. Reads having Phred Score(Q) < 30 and over-represented sequences i.e. primer sequences, adapter sequences were removed using Trimmomatic (53) (http://www.usadellab.org/cms/?page=trimmomatic) from both pairs. For binding events comparison, PU.1 (0 h) and Irf4 (0 and 2 h) ChIP-seq data of bone marrow-derived dendritic cell (BMDC) provided with LPS stimulation were accessed form GSE36104 (54).

### RNA-seq analysis

Filtered reads from transcriptome data were aligned with Tophat2 (55) using mm10 mouse genome assembly and Gencode M17 (GRCm38.p6) as reference transcript file. Rest of the alignment parameters remained same as Tophat2 is optimized for mammalian sequence alignment by default. Cufflinks v2.2.1 was used to calculate the abundance of transcripts from aligned files in terms of fragments per kilobase per million of reads (FPKM) and also generated the assembly file for differential expression using CuffDiff. All the assembly files from biological replicates were merged using Cuffmerge command from the same tool. Genes/transcripts form differential expression analysis having *p* < 0.05(significance) and *q* < 0.05 (false discovery rate) were classified as significant.

### ChIP-seq analysis

Alignment of ChIP-seq data was aligned with Bowtie2 (56) using mm10 mouse genome assembly as reference genome and—no-mixed,—no-discordant options to avoid unpaired read alignments. Aligned reads were deduplicated using Samtools (57) and randomly down-sampled to 22 million reads using Picard tool (https://broadinstitute.github.io/picard/). Peak calling was done using find Peaks tool form Homer suite (58) using a threshold of 4-fold change against input control and–factor option. Transcription factor motifs in −100/+100 region of the peaks were searched using findMotifsGenome.pl, and peaks were annotated to nearby gene using annotated Peaks.pl. Motifs obtained from both “*De novo*” and “Known Motifs” (having highest motif score) search having *p* < 10-20 were considered significant. Aligned reads from BMDC PU.1 and Irf4 ChIP-seq were compared with Zeb1 ChIP-seq data using SeqMiner (59) k-means clustering. To validate the ChIP-seq results we randomly selected Zeb1 peaks found in ChIP-seq to confirm the enrichment using two independent ChIP-qPCR experiments.

### Pathway analysis

Ingenuity Pathway Analysis (IPA) software from Qiagen was used throughout the analysis process and *p*-value cutoff of 0.05 was considered significant. All the raw results of pathway analysis are attached as Supplementary Files.

### Visualization

Gene expression scatter plot, pathway bar plots and other sequencing data representing bar graphs were generated using Ggplot2 (https://cran.r-project.org/web/packages/ggplot2/index.html) R package. The heatmap was generated using Complex Heatmap R package with row-wise clustering option (60).

## Results

### EMT factor Zeb1 KD suppresses activation, Co-stimulation and cytokine production in CD8α^+^cDC1 DCs

**Figure 1:**
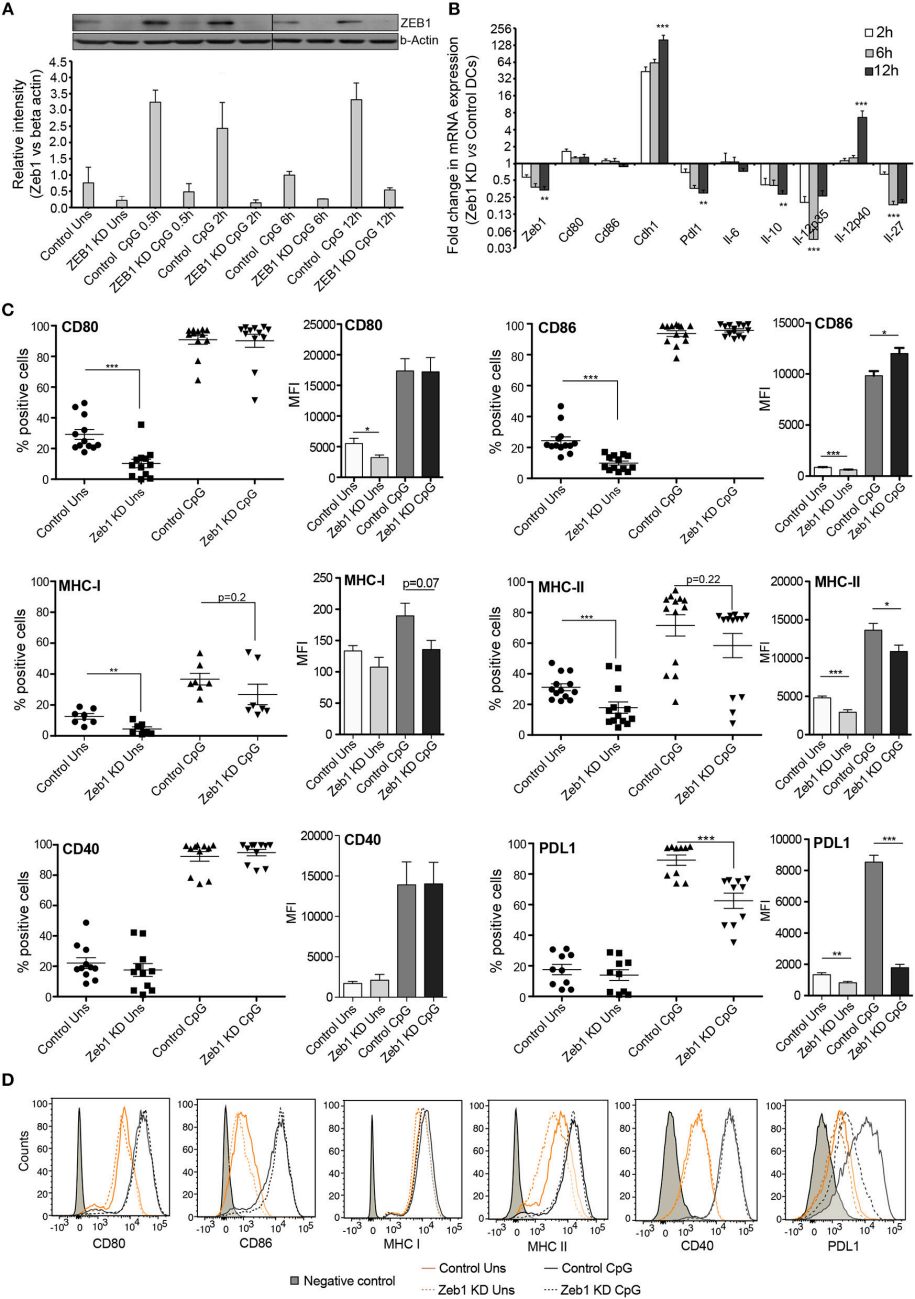
Zeb1 transcription factor KD suppresses activation and co-stimulation of CD8α^+^ cDC1 MutuDCs. **(A)** Western blot and its densitometric analysis showing the ZEB1 protein expression kinetics and its KD by shRNA at 0, 0.5, 2, 6, and 12 h after CpG activation in CD8α^+^ cDC1 DCs *n* = 2. **(B)** Bar-plot demonstrating the fold changes in the transcript expression of DC activation/co-stimulation markers and selected cytokine genes in Zeb1 KD DCs compared to control cells using qPCR *n* = 3. **(C)** Scatter-plots showing the percentage positive cells for cell-surface expression of activation and co-stimulatory markers CD80, CD86, MHC-I, MHC-II, CD40, and PDL1 on Zeb1 KD cDC1 MutuDCs compared to control cells before and after 12 h CpG activation. The corresponding panels with bar plots depict the Median Florescence Intensity (MFI) shifts for the respective genes *n* = 6–12. **(D)** Representative histogram plots demonstrating the MFI shift observed for the activation/co-stimulatory markers and cytokines. *p*-values are calculated using two tailed unpaired student's *t*-test, error bars represent SEM. * ≤ 0.05, ** ≤ 0.01, *** ≤ 0.001.

In this study, we have used a CD8α^+^ cDC1 MutuDC line recently developed and well characterized by Fuertes Maracco and colleagues (9). They showed that this DC line mimic remarkably the primary lymphoid resident cDC1 responses isolated from spleen. While analyzing an unpublished RNA-seq data from these cDC1 cells, we found that Zeb1 transcript is constitutively expressed in cDC1 and upon TLR9 stimulation by CpG the expression increased significantly (Supplementary Figures 1A,B). To characterize the functional role of Zeb1 in cDC1, we generated a stable Zeb1 KD in cDC1 MutuDC line using lentiviral shRNA transduction followed by puromycin selection. We used three different shRNAs targeting different regions of Zeb1 transcript and found that two of them showed significant depletion of Zeb1 transcript (Supplementary Table 8). For downstream analyses we moved ahead with one shRNA sequence i.e., shRNA3, which showed higher Zeb1 depletion. We obtained 55–80% reduction of Zeb1 transcript in stable KD DCs and a concomitant decreased ZEB1 protein levels before and after 0.5, 2, 6, and 12 h after CpG activation as evident from western blotting analysis (Figures 1A). The western blot analysis demonstrated low levels of ZEB1 expression in unstimulated cells and a prominently increased expression within first few hours of CpG challenge that was decreased at later time points i.e., 6 and 12 h (Figures 1A,B).

**Figure 2:**
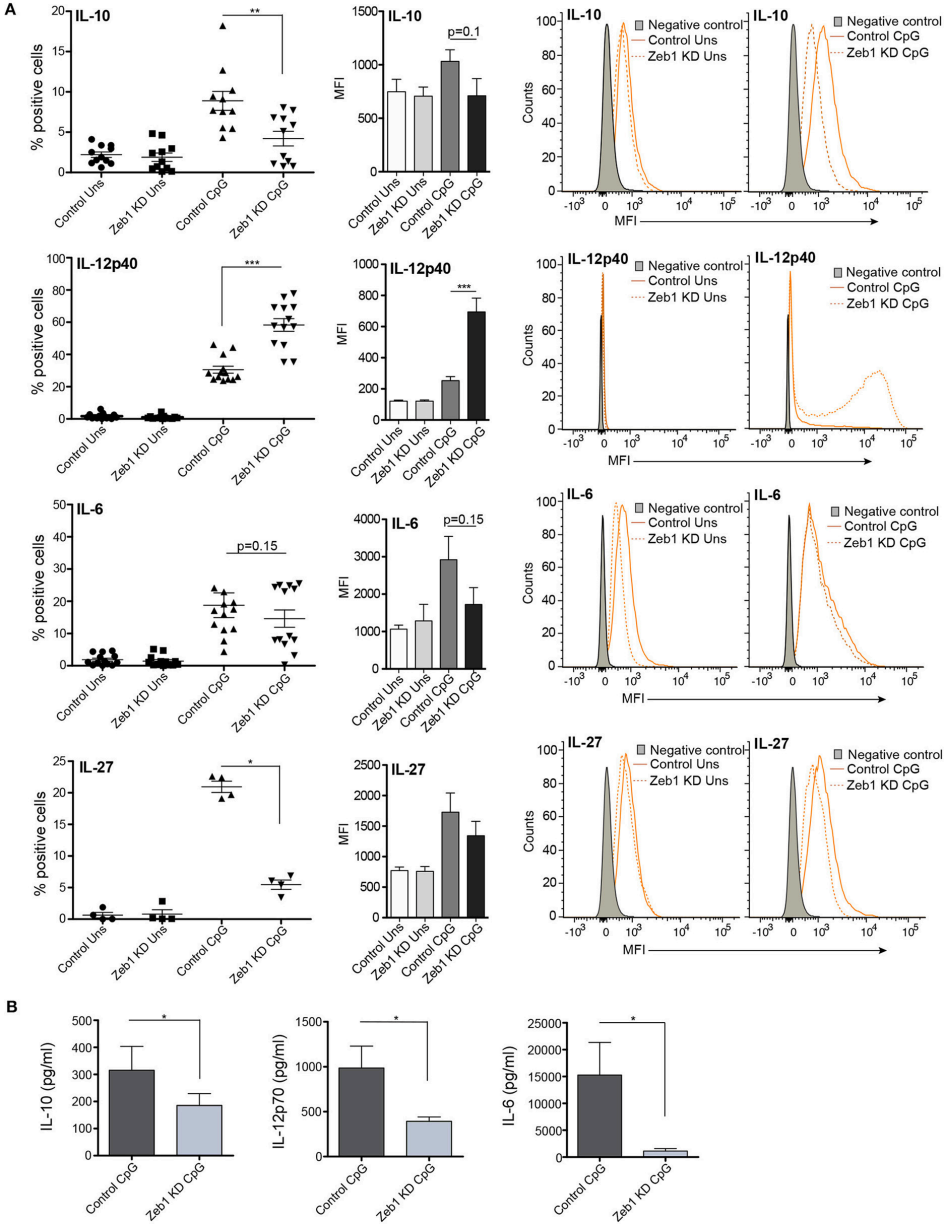
EMT factor Zeb1 KD suppresses expression of important DC response cytokines in cDC1 MutuDCs. **(A)** Scatter-plots showing the percentage positive cells for intracellular cytokines IL-10, IL-12p40, IL-6, and IL-27 in Zeb1 KD cDC1 MutuDCs compared to control cells before and after 12 h CpG activation. The corresponding bar plots and representative histograms in each panel depicting the MFI shifts for these cytokines in Zeb1 KD as compared to control DCs *n* = 8–12. **(B)** Bar-plots demonstrating the Bio-plex based quantitation of IL-10, IL-12p70, and IL-6 cytokines secreted in the supernatants of 12 h CpG activated Zeb1 KD and control DCs *n* = 5. *p*-values are calculated using two tailed unpaired student's *t*-test, error bars represent SEM. * ≤ 0.05, ** ≤ 0.01, *** ≤ 0.001.

After confirming the Zeb1 KD at transcript and protein level in stable KD DCs, we analyzed the impact of its depletion on DC activation and concomitant cytokine expression in CD8α^+^ cDC1 MutuDCs. We performed detailed immune profiling of these stable Zeb1 KD DCs before and after 2, 6, and 12 h of CpG stimulation. It has been well established that Zeb1 directly represses Cdh1 gene and therefore first we analyzed the transcript expression of Cdh1 gene and found it to be significantly increased after Zeb1 depletion in cDC1 DCs confirming the impact of Zeb1 depletion (Figure 1B). Then we investigated the impact of Zeb1 KD on DC activation and co-stimulation along with expression of cytokine genes 12 h after CpG challenge using qPCR, flow cytometry (FACS) and multiplex ELISA (Bioplex) to profile the Zeb1 mediated immune-modulations. We found that Zeb1 KD DCs showed significantly reduced transcript expression of important DC response cytokines Il-10 and Il-27, while Il-12 (subunit p40) showed significant and sustained increase after 12 h of CpG stimulation in Zeb1 KD DCs (Figure 1B). Moreover, the Il-12p35 subunit of IL-12 cytokine showed significantly decreased expression after Zeb1 KD (Figure 1B). We did not observe any significant change in the mRNA expression of Cd80 and Cd86 activation markers upon Zeb1 KD before and after CpG activation (Figure 1B). FACS analysis showed significantly decreased expression of CD80 and CD86 in Zeb1 KD unstimulated DCs as compared to control cells, but no significant differences were observed after CpG stimulation (Figure 1C). The MFI analysis showed a significant increase in CD86 in CpG condition, whereas percent positive cells showed an insignificant increasing trend as CpG activation makes nearly 99–100 percent cells positive for CD86 (Figure 1C). The CD40 expression was unchanged in Zeb1 KD as compared to control DCs, whereas MHC-I and MHC-II percent positive cells showed a significant decrease in unstimulated condition (Figures 1C,D). In addition, the MFI shifts depicted a significant MHC-II decrease before and after CpG activation with a decreasing trend for MHCI (Figures 1C,D). Consequently the intracellular levels of IL-10 and IL-27 cytokines were significantly decreased in Zeb1 KD DCs after CpG activation (Figures 2A,B). Moreover, the MFI analysis (bar-plots and histograms) also showed similar trends (Figures 1C,D, 2A). The cytokine IL-6 showed insignificant but decreasing trend (Figure 2A). Besides, the IL-12p40 levels were significantly increased in 12 h CpG activated Zeb1 KD DCs. At early time points (2 and 6 h) of CpG activation, we did not observe any significant change in the IL-12p40 expression (data not shown). Furthermore to estimate the secreted cytokine levels, we performed multiplex ELISA i.e., Bio-plex analysis, which demonstrated a significant decrease in IL-6, IL-10, IL-12p70, and IFNγ cytokine levels in CpG activated Zeb1 KD DCs (Figure 2B and Supplementary Figure 1F). We observed significantly decreased levels of IL-12p70 in the culture supernatants of 12 h CpG activated Zeb1 KD DCs in contrast to a significant increase we found for IL-12p40, an important subunit of this cytokine (Figure 2B). This may be due to significantly decreased IL-12p35 subunit, which is an exclusive subunit of bioactive inflammatory cytokine IL-12. Other important DC markers like PDL1 and IL-27 also showed a significant decrease after Zeb1 depletion as compared to control DCs (Figures 1C, 2A). These results showed that Zeb1 depletion suppressed DC activation and co-stimulation leading to decreased secretion of important DC response cytokines.

The cDC1 MutuDCs that we employed in our study express high levels of both TLR3 and TLR9 receptors (9). Therefore, we also treated Zeb1 KD and control DCs with TLR3 ligand pIC and CpG + pIC simultaneously to activate both TLR3 and TLR9 receptors together. We found that pIC resulted in weak activation of DCs as compared to CpG whereas simultaneous CpG + pIC activation resulted in strong activation as evident from CD80 and CD86 expression and synergistic expression of cytokines like IL-6, IL-10, and IL-12p40 (Supplementary Figures 1C,D). We found that Zeb1 depletion resulted in similar decrease in activation, co-stimulation and cytokine genes as we found after CpG activation. These results further confirmed that Zeb1 KD results in suboptimal activation of DCs leading to decreased expression of pro- and anti-inflammatory cytokines in cDC1 DCs, irrespective of any strong antigenic challenge.

**Figure 3:**
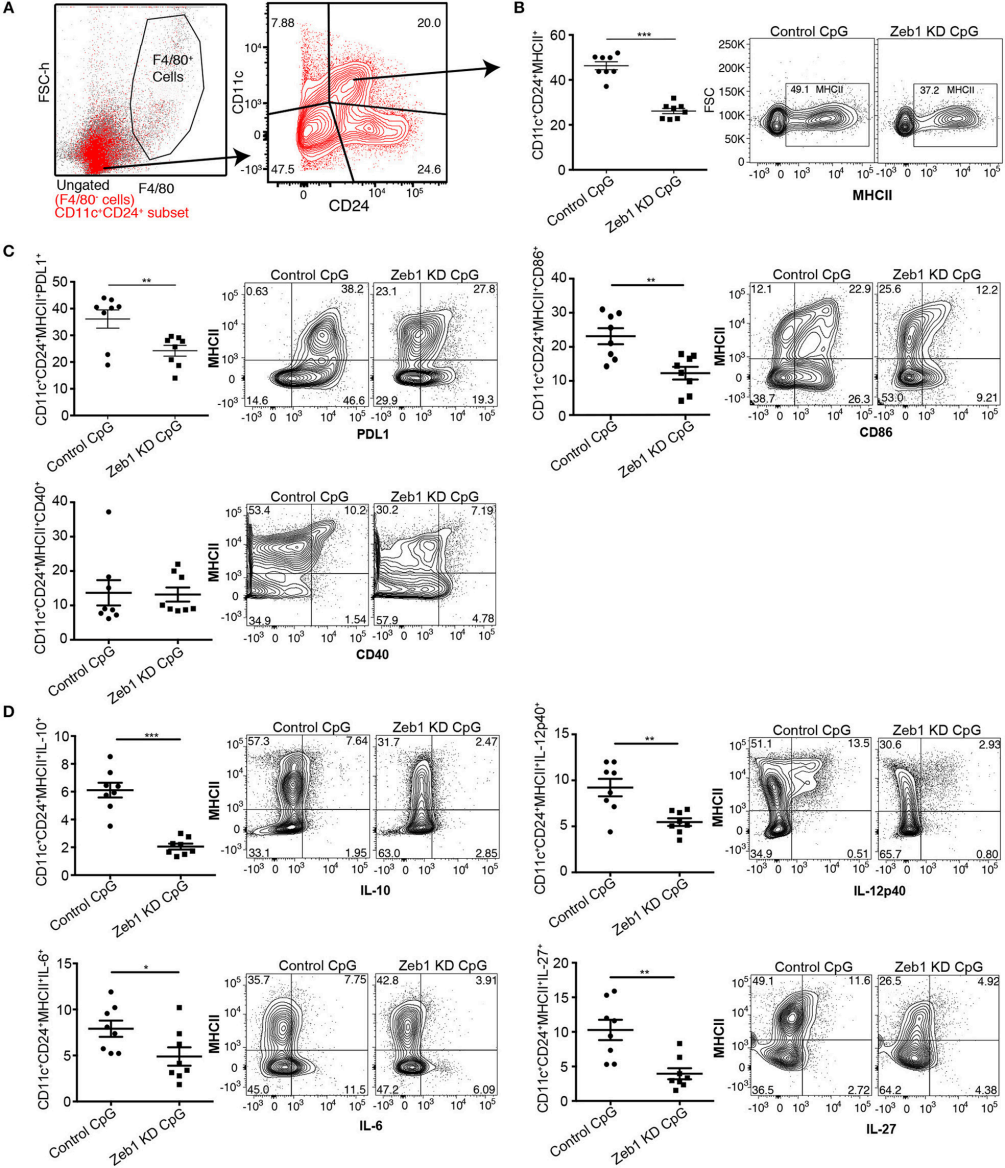
Zeb1 depletion in bone-marrow derived primary cDC1 DCs showed decreased DC activation, co-stimulation and cytokine secretion *n* = 6–8. **(A)** FACS contour-plot showing the gating strategy used to remove F4/80 positive macrophage population from the CD11c^+^ DCs to analyze the impact of Zeb1 KD on CD11c^+^CD24^+^MHCII^+^ DC population. High CD11c^+^CD24^+^ double positive DCs were considered as cDC1 DCs for analysis. **(B)** Scatter-plot and representative contour plots depicting the percentage of MHCII positive cells in F4/80^−^CD11c^+^CD24^+^ gated BMDCs treated with control and Zeb1 shRNA3 followed by 12 h CpG stimulation *n* = 6–8. **(C)** Scatter-plots and representative contour plots depicting the percentage positive cells for cell surface markers PDL1, CD86, and CD40 in F4/80^−^CD11c^+^CD24^+^ gated BMDCs treated with control and Zeb1 shRNA3 followed by 12 h CpG stimulation *n* = 6–8. **(D)** Scatter-plots showing the percentage positive cells for intracellular cytokines IL-10, IL-12p40, IL-6, and IL-27 in F4/80^−^CD11c^+^CD24^+^MHCII^+^ gated BMDCs treated with control and Zeb1 shRNA3 followed by 12 h CpG stimulation *n* = 6. *p*-values were calculated using two tailed unpaired student's *t*-test, error bars represent SEM. * ≤ 0.05, ** ≤ 0.01, *** ≤ 0.001.

To validate our *in vitro* findings, we generated primary cDC1 DCs from the bone marrow precursor cells using fms-like tyrosine kinase 3 ligand (FLT3L) supplemented medium (see Methods for details) (47). Bone marrow culture with FLT3L is the recent method that allow the generation of both cDCs and pDCs. In purview of published reports, which suggests that FLT3L derived DCs (FL-DCs) are CD11c^hi^CD24^hi^ and CD11b^low^, are putative of CD8α^+^ cDC1 equivalent (47, 61), therefore we gated CD11c^+^CD24^+^MHCII^+^ population in FACS to analyze the impact of Zeb1 transient depletion on cDC1 activation and cytokine expression. We found that transient KD of Zeb1 in primary cDC1 cells using Zeb1 shRNA reduced their activation, co-stimulation and production of cytokines like IL-6, IL-10, IL-12p40, and IL-27 as compared to control KD DCs (Figures 3A–D and Supplementary Figure 2A). In contrast to Zeb1 KD MutuDCs, IL-12p40 showed decreased expression in BMDCs. This could be due to transient Zeb1 KD, differential expression kinetics of IL-12p40 in BMDCs or due to cell heterogeneity of BMDCs. We also found that Zeb1 is expressed at similar levels in different primary DC subsets cDC1, cDC2, and pDCs isolated from spleen of FLT3L transgenic mice. Here we have focused on the role of Zeb1 in cDC1 DCs but it would be further intriguing to explore its importance in other DC subsets as well (Supplementary Figure 2F).

### OT-II T helper cells Co-cultured with Zeb1 KD DCs enhanced Th2 responses

**Figure 4:**
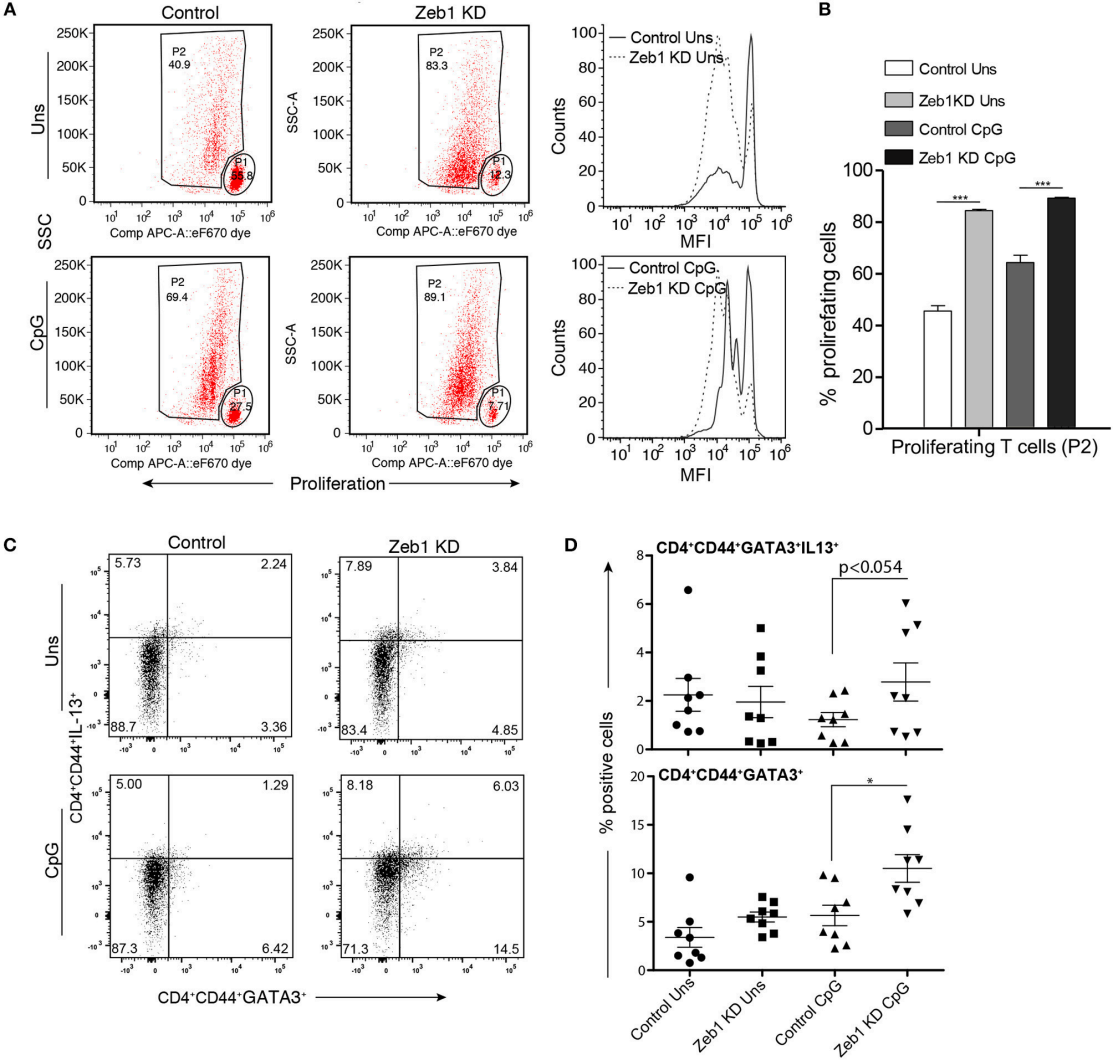
OT-II T helper cells co-cultured with CpG activated Zeb1 KD cDC1 MutuDCs showed enhanced proliferation and differentiation toward Th2 phenotype. **(A)** Representative FACS dot-plots showing gated parent population (P1) and proliferating cell population (P2) depicting the changes in proliferation rate of OT-II Th cells co-cultured for 72 h with Zeb1 KD cDC1 MutuDCs as compared to control DCs with or without CpG pulsing. The corresponding panel shows the histogram showing the MFI shifts for the proliferation of T cells. *n* = 3. **(B)** Bar-plot depicting the percentage of proliferating Th cells (P2) in CD4^+^ T cells co-cultured with unstimulated and CpG activated Zeb1 KD and control DCs *n* = 3. **(C)** FACS dot-plots showing the percentage positive CD4^+^CD44^+^GATA3^+^IL-13^+^ Th cells in Th cells co-cultured with unstimulated and CpG pulsed Zeb1 KD and control cDC1 MutuDCs for 96 h *n* = 8. **(D)** Scatter-plots showing the percentage of double positive cells for GATA3^+^IL-13^+^ and single positive GATA3^+^ cells in effector CD4^+^CD44^+^ Th cell population *n* = 8. *p*-values are calculated using two tailed unpaired student's *t*-test, error bars represent SEM. * ≤ 0.05, *** ≤ 0.001.

As we found a consistent decrease in all the measured cytokines in Zeb1 KD DCs we were interested to identify if Zeb1 KD cDC1 MutuDCs would functionally interfere in T-helper (Th) cell differentiation. For the same, Zeb1 KD and control DCs were pulsed with OTII peptide with or without CpG for 2 h. Then, CD4^+^ Th cells isolated from spleen of OT-II transgenic mice were labeled with a proliferation dye efluor 670 followed by co-culture with these DCs for 72 h. Upon priming by DCs, the naïve T helper cells first undergo several rounds of clonal amplification and then polarization into various effector subtypes depending upon the DC responses (62, 63). We found that Zeb1 KD DCs induced higher antigen-specific CD4^+^ Th cell proliferation compared to control DCs (Figure 4A). This was also observed when DCs had been previously stimulated with CpG, which induced even higher T cell proliferation after 3 days of co-culture (Figures 4A,B). As we observed a decrease in activation and co-stimulation markers in Zeb1 KD DCs the increased proliferation of T cells was puzzling and therefore we looked into expression of IL-2 in cell culture supernatants of Zeb1 KD and control cells by Bio-plex. We found increased IL-2 cytokine after CpG activation in Zeb1 KD DC supernatants as compared to control cells (Supplementary Figure 2E). In addition, we looked into T cell differentiation profiles of these co-cultured Th cells. We found significantly increased differentiation of Zeb1 KD DCs primed Th cells toward Th2 subtype marked by increased number of GATA3 and GATA3^+^IL-13^+^ expressing Th cells (Figures 4C,D). At the same time the Tbet^+^IFNγ^+^ expressing Th1 cells were majorly decreased in CpG activated Zeb1 KD condition (Supplementary Figures 2B,C). We did not observe any significant difference in the Treg differentiation marker FoxP3 (Supplementary Figure 2D) In unstimulated conditions we did not found any significant increase in GATA3^+^ alone or GATA3^+^IL-13^+^ double positive Th cells (Figures 4C,D) and on the contrary the Tbet^+^IFNγ^+^ population was increased (Supplementary Figures 2B,C)

Moreover we also performed allogeneic mixed lymphocytic reaction (MLR) assay to confirm the induction of Th2 responses by Zeb1 KD DCs (Supplementary Figure 3A). We found that the CD3^+^CD4^+^CD44^+^ effector Th cells showed enhanced Th2 responses as evidenced by significantly increased GATA3 and IL-4 expression in T cells primed by CpG pulsed Zeb1 KD compared to control DCs (Supplementary Figure 3B). On the other hand, the Th1 subtype polarization marker Tbet and its signature cytokine IFNγ showed insignificant but decreasing trend (Supplementary Figure 3C). These analyses demonstrated that Zeb1 has the potential to modulate T helper cell differentiation toward Th2 subtype by modulating DC responses. We also looked into the impact of Zeb1 KD cDC1 on CD8^+^ T cell function by gating them separately from CD4^+^ Th cells (Supplementary Figure 3D). We found that the effector CD8^+^ T cells generated in co-culture with Zeb1 KD DCs produced significantly less Granzyme and Perforin as compared to CD8^+^ T cells cultured with control DCs (Supplementary Figures 3D,E) suggesting Zeb1 as important factor for inducing optimal T cell cytotoxic responses.

### Adoptive transfer of Zeb1 depleted DCs enhanced helminth clearance in mice

**Figure 5:**
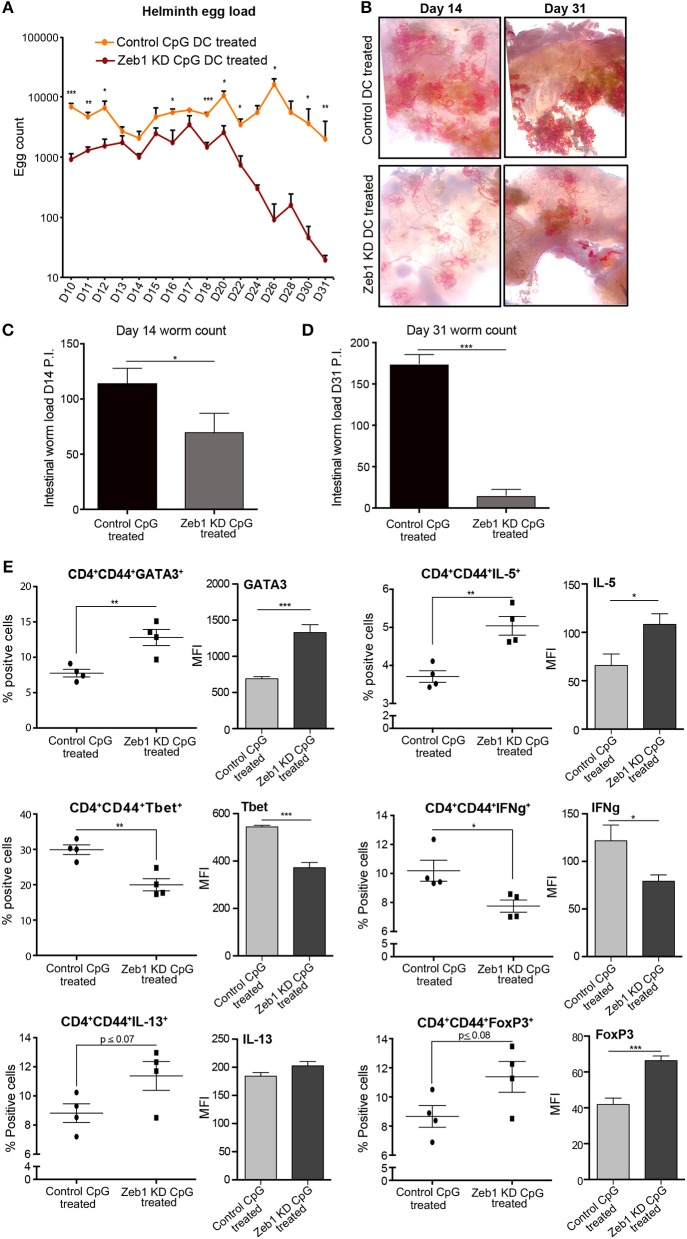
Adoptive transfer of CpG activated Zeb1 KD cDC1 MutuDCs in helminth-infected mice enhances worm clearance by increasing Th2 responses. **(A)** Line-graphs depicting the Helminth egg counts in the feces of CpG pulsed Zeb1 KD CD8α^+^ cDC1 MutuDCs and control DC treated mice from D10 to D31. The mice were infected with *H. polygyrus* larvae at D0 and treated with CpG pulsed control and Zeb1 KD CD8α^+^ cDC1 cells consecutively at D7, D9, and D11. The egg counting was started from D10 till D31. At day 9 there were no eggs detected in feces of animals *n* = 10. **(B)** Representative intestinal sections of helminth infected mice at D14 and D31 after helminth infection and treatment with CpG pulsed Zeb1 KD and control DC treated animals *n* = 5. **(C)** Intestinal worm load at day 14 in mice adoptively treated with CpG activated Zeb1 KD and control DCs *n* = 5. **(D)** Intestinal worm load in mice adoptively treated with CpG activated Zeb1 KD and control DCs at day 31 after infection *n* = 5. **(E)** Scatter-plots for Th1, Th2, and Treg markers like GATA3, IL-5, Tbet, IFNγ, IL-13, and FoxP3 from detailed immune profiling of CD4^+^CD44^+^ effector Th cells isolated form mesenteric lymph nodes of helminth infected animals and treated with activated Zeb1 KD and control DCs. The corresponding panels also depict bar plots for MFI shifts for each marker in CD4^+^CD44^+^ effector Th cell population *n* = 5. *p*-values are calculated using two tailed unpaired student's *t*-test, error bars represent SEM. * ≤ 0.05, ** ≤ 0.01, *** ≤ 0.001.

After confirming that Zeb1 KD cDC1 MutuDCs enhanced Th2 cell development *in vitro*, we were interested in determining if these DCs were able to affect Th2 responses in an *in vivo* parasite infected animal model. Helminth (*Heligmosomoides polygyrus*) infection mice model is considered as one of the best-characterized disease models where it has been established that perturbation of Th cell subtype responses modulates the worm load in the intestine (64–67). Besides that the penetrance of pathogenesis is quite uniform in this disease model. Therefore, we performed adoptive transfer of CpG pulsed control and Zeb1 KD MutuDCs in *H. polygyrus* infected mice to identify its physiological impact on disease burden. The egg load in the feces were calculated starting from day 9 (D9) till 1 month after adoptive transfer of DCs to get an idea about the mature worm abundance in the intestine of infected animals. At D9, there were no eggs observed in both Zeb1 KD and control DC treated animals. This observation is consistent with the previous reports that worms get mature and move to intestinal lumen on the D10 and reproduce, resulting in release of eggs in feces (68). Interestingly at D10, we found that Zeb1 KD DC treated animals showed less egg counts in their feces compared to control DC treated animals even after injecting them with equal number of L3 stage larvae at D0 (Figure 5A). In addition, in Zeb1 KD DCs treated mice the intestines were almost clear of helminth infection as no or only few worms were found in the intestine depicted by the drastically reduced number of eggs on D31 (Figure 5B). Five animals from each group were dissected at D14 and D31 to estimate the worm load in the intestines. We found that animals treated with CpG activated Zeb1 KD DCs showed a significant decrease in intestinal helminth load as compared to control DC treated animals at D14 (Figure 5C). Besides, at D31 insignificant differences were observed in intestinal helminth count in animals treated with unstimulated Zeb1 KD and control DCs as clear from similar egg counts found at D31 (data not shown). However, the animals treated with CpG pulsed Zeb1 KD DCs were almost free of helminth infection in the intestine as compared to control DC treated mice at D31 (Figure 5D).

Furthermore to identify the impact of CD8α^+^ cDC1 MutuDCs adoptive transfer on Th cell polarization in animals leading to the observed phenotype, we performed detailed Th cell subtype profiling from mesenteric lymph nodes (MLNs) of all the infected and treated animals at D14 and D31. We found that in CpG pulsed Zeb1 KD DC treated animals at D14 presented increased number of Th2 effector CD4^+^CD44^+^ cells with significantly higher expression of GATA3 and IL-5 as compared to control DC treated animals (Figure 5E). The cytokine IL-13 also showed an insignificant but increasing trend (Figure 5E). We also observed mice that were treated with CpG pulsed Zeb1 depleted DCs had reduced Tbet and IFNγ positive cells in the MLNs. FoxP3, a Treg marker showed an increase in Zeb1 KD cells, which is well reported to be elevated during Th2 response (Figure 5E). At D31, we found a significant increase of IL-5 and IL-10. The cytokine IL-13 showed increasing trend, whereas IFNγ was significantly increased (Supplementary Figure 4), which could be the reason for increased Tregs. Increased Th2 cells in MLNs of helminth infected animals by treating animals with Zeb1 KD DCs strongly suggested that Zeb1 depletion in CD8α^+^ cDC1 could potentiate Th2 responses *in vivo*, affecting helminth clearance.

### Transcriptome analysis of Zeb1 KD DCs showed an enrichment of Th2 pathway

**Figure 6:**
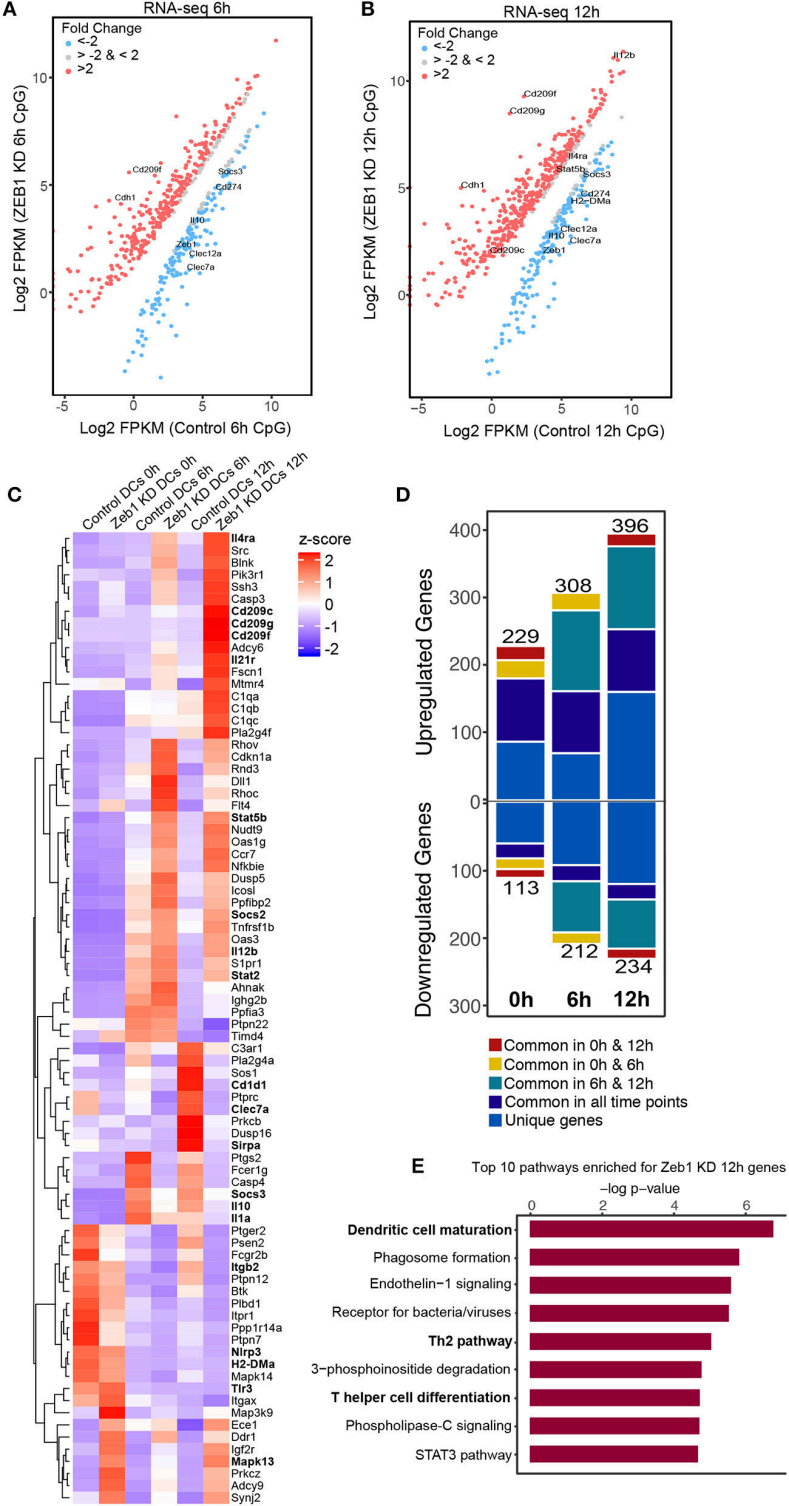
Global transcriptome analysis of Zeb1 KD cDC1 MutuDCs showed enrichment of pathways involved in T helper cell differentiation and Th2 pathways. Two independent biological replicates with three time points (0, 6, 12 h) were used for this analysis. **(A)** RNA-seq scatter-plot showing the differentially regulated genes in Zeb1 KD cDC1 MutuDCs as compared to control DCs at 6 h after CpG activation *n* = 2. **(B)** Scatter-plot showing the differentially regulated genes in Zeb1 KD cDC1 MutuDCs as compared to control DCs at 12 h after CpG activation *n* = 2. **(C)** Heat-map generated from a list of manually curated genes identified from differentially regulated gene list in Zeb1 KD RNA-seq data, which includes the genes that are reported to induce Th2 responses upon activation by DCs. Major DC response genes found to be predominantly regulated after Zeb1 KD are highlighted in bold. To generate this bar-graph Z-score was calculated for each gene from normalized FPKM values to demonstrate the differential regulation of genes in control and Zeb1 KD DCs at 0, 6, and 12 h after CpG activation. **(D)** Bar-plot depicting the number of genes differentially regulated in Zeb1 KD DCs at 0, 6, and 12 h CpG activation as compared to control DCs. Different color codes indicate the genes that are unique or similar in the list of differentially regulated genes at different time points (0, 6, and 12 h). **(E)** Bar-plot depicting the biological pathways that were significantly enriched for the genes that differentially regulated after Zeb1 KD in 6 and 12 h CpG activation as compared to control cells. Ingenuity Pathway Analysis (IPA) was used to perform pathway enrichment analysis.

To understand mechanisms underlying the control of Zeb1 mediated DC responses we performed RNA-seq analysis of control and Zeb1 KD cDC1 MutuDCs at 0, 6, and 12 h after CpG stimulation. First we confirmed the significantly decreased transcript levels of Zeb1 and a concomitant significant increase in Cdh1 expression in RNA-seq datasets at all the analyzed time points (Supplementary Table 1). Downstream analysis and manual curation of the genes that were differentially expressed (corrected *p*-value ≤ 0.05) in Zeb1 KD DCs showed a significant down-regulation of cytokines like Il-6, Il-10, and Il-27 along with several C-type lectin receptors (CLRs) such as Clec1a, Clec4a1, Cleca7A (Dectin-1), Clec9a and Clec12a as compared to control cells (Figures 6A,B and Supplementary Table 1). It has been reported that CLRs present on DCs act in a pathogen/antigen dependent manner to control Th cell differentiation. Whereas one of the CLR i.e., DC-SIGN (Cd209c, Cd209f, Cd209g) showed significant and highest increase in 6 h as well as 12 h CpG activated Zeb1 KD DCs as compared to control cells (Figures 6A–C and Supplementary Table1). We found that there were more upregulated genes after Zeb1 KD as compared to down-regulated ones at all the time points suggesting toward global repressive function of Zeb1 (Figure 6D). There were 229, 308, and 396 genes upregulated after Zeb1 KD at 0, 6 and 12 h time point, whereas 113, 212, and 234 genes were down regulated respectively (Figure 6D and Supplementary Table 1). Moreover, the major anti-inflammatory or tolerogenic cytokine Il-10 was also significantly decreased at both 6 and 12 h time points in Zeb1 depleted cells (Figure 6C and Supplementary Table 2). Ultimately to identify the biological pathways that were enriched for the genes differentially expressed in Zeb1 KD DCs after 6 and 12 h CpG activation, we performed Ingenuity pathway analysis (IPA). We found that at both the time points there was enrichment of “Dendritic cell maturation,” “Receptors for bacterial/virus recognition,” “Th2 pathways,” “Th cell differentiation,” and “STAT3 pathways” (Figure 6E, Supplementary Figures 5A,B and Supplementary Table 3).

### Zeb1 chip-seq identified its direct and indirect target genes in CD8α^+^ cDC1 MutuDCs

**Figure 7:**
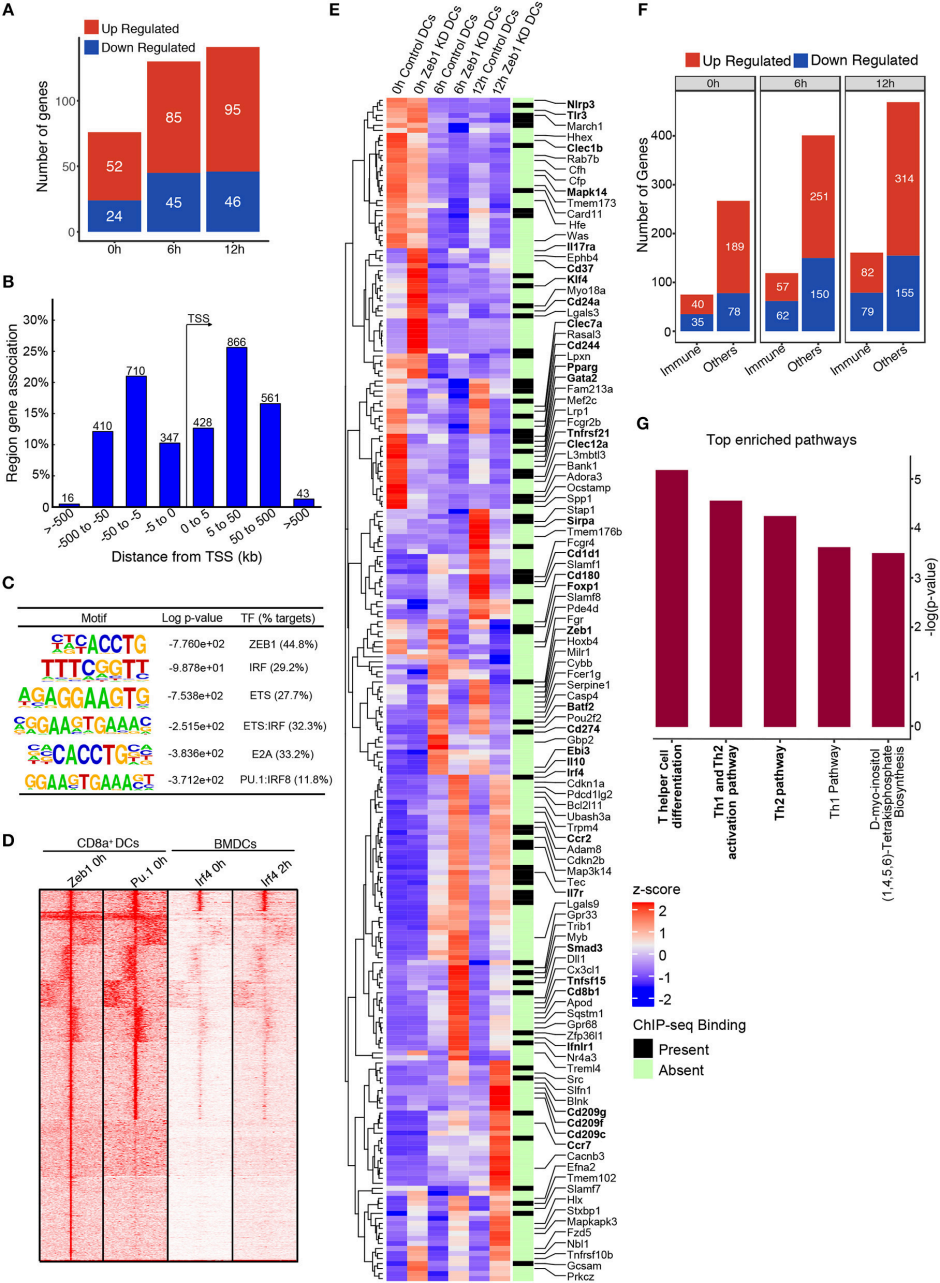
Integrative genomic analysis using ChIP-seq and RNA-seq identified that Zeb1 does not act as global repressor of immune response genes. **(A)** Bar-plot depicting the genes that were directly regulated (upregulated and down-regulated) by Zeb1 KD at 0, 6, and 12 h after CpG activation in cDC1 DCs. The direct target genes of Zeb1 were identified by correlating the gene list from Zeb1 KD RNA-seq and Zeb1 ChIP-seq binding analysis. **(B)** Genomic Regions Enrichment of Zeb1 ChIP-seq bound peaks by annotations tool GREAT showing the genomic locations of Zeb1 bound regulatory regions with respect to gene transcription start sites (TSS). **(C)** List of top *de-novo* motifs significantly enriched at the Zeb1 bound peaks in unstimulated DCs and their annotated TFs. Zeb1 motif was found to be the top highly enriched motif. **(D)** SeqMINER clustering demonstrating the overlap of Zeb1 bound peaks in unstimulated CD8α^+^ cDC1 with PU.1 bound genomic regions in similar condition. The peaks were also overlapped with IRF4 ChIP-seq publicly available data of control and LPS stimulated primary BMDCs. **(E)** Heat-map demonstrating the immune response pathway genes that were differentially regulated after Zeb1 KD 0, 6, and 12 h after CpG activation as compared to control cells. The ChIP-seq binding was also overlapped to identify if Zeb1 directly regulates immune response genes. All the immune response genes that were differentially regulated (>2-fold up or down-regulated) are listed. The genes that were reported and are important in Th cell polarization through DCs are highlighted in bold. To generate this bar-graph Z-score was calculated for each gene from normalized FPKM values to demonstrate the differential regulation of genes in control and Zeb1 KD DCs at 0, 6, and 12 h after CpG activation. **(F)** Bar-plot depicting the differential regulated genes in Zeb1 KD DCs at 0, 6, and 12 h after CpG activation. The genes were classification into two different groups based on GO annotation i.e., immune response genes (immune) and other pathway genes (others). It was observed that in immune response gene group there is preferential upregulation of genes after Zeb1 KD whereas in the other group there was higher number of upregulated genes (>1.6- to 2.5-fold) compared to down-regulated ones. **(G)** Bar-plot showing the IPA pathways significantly enriched for the genes that were directly bound and regulated by Zeb1 at 12 h after CpG activation.

We performed ChIP-seq for Zeb1 in unstimulated DCs to identify the genes that were directly bound and regulated by Zeb1. The major aim was to correlate the Zeb1 binding in ChIP-seq with RNA-seq to identify the genes that were directly controlled by Zeb1 in DCs. We found ~3400 genomic regions bound by Zeb1 in unstimulated CD8α^+^ cDC1 (Supplementary Table 4). Upon overlap of genes bound in ChIP-seq with RNA-seq list we found 76, 130, and 141 genes to be directly regulated by Zeb1 (Figure 7A). Out of these genes 52, 85, and 95 were upregulated, whereas 24, 45 and 46 genes were down regulated at 0, 6, and 12 h respectively after Zeb1 KD (Figure 7A). To identify the Zeb1 bound genomic regions with respect to transcription start site (TSS), we did GREAT analysis and found that majority of Zeb1 bindings were distal (775 peaks in ±5 KB, 1576 peaks in >±5 KB and < ±50 K region) and far from TSS (Figure 7B). Then, to identify if Zeb1 DNA binding motif is enriched on these bound peaks we did *de novo* motif analysis using HOMER and found 42% of bound regions showed canonical Zeb1 motif whereas other Zeb1 bound genomic regions showed significant enrichment of DNA motifs for IRF, ETS (PU.1), E2A and ETS-IRF (PU.1-IRF) TFs (Figure 7C). To experimentally validate it, we performed ChIP-seq for PU.1, and the SeqMINER overlap with Zeb1 peaks showed that indeed PU.1 overlaps strongly at Zeb1 bound genomic regions (Figure 7D). We also overlapped publicly available IRF4 ChIP-seq data from unstimulated and 2 h LPS stimulated BMDCs with Zeb1 and found that IRF4 also showed similar percentage of overlap as predicted in our *de novo* motif analysis (Figures 7C,D and Supplementary Table 5). Moreover, Th1 and Th2 pathways are enriched for genes annotated to Zeb1-PU1-IRF4 overlapping genomic regions (Supplementary Figure 5C). Although Zeb1 is a known transcriptional repressor and there are more upregulated genes as compared to down regulated genes in RNA-seq data, we observed that most of the DC immune response genes were down regulated after Zeb1 depletion in our *in vitro* and *ex vivo* immune-profiling experiments. To understand it better, we divided all the Zeb1 KD differentially regulated genes into two groups based on gene ontology, i.e., immune response pathway and the other pathway genes and looked for any preferential Zeb1 bindings differences in these two groups. Surprisingly, the ratio of Zeb1 unbound vs. bound regulated genes was ≥2.5 for both the gene groups. Then, we analyzed the number of up- vs. down-regulated genes in immune response and other pathway group and interestingly we found that similar number of immune response genes were differentially regulated (ratio of ~1.0 for up- vs. down genes) whereas for other pathways gene group there were more upregulated genes (ratio ≥ 1.6–2.5) (Figures 7E,F and Supplementary Table 6). This suggested that Zeb1 does not appear to be a global transcriptional repressor for immune response genes. Ultimately we analyzed the pathways enriched for the genes directly bound and regulated by Zeb1 using IPA. We found “T helper cell differentiation” and “Th2 pathway” to be significantly enriched for genes differentially regulated at 12 h time point (Figure 7G and Supplementary Table 7). It was interesting to find that even with such a low number of direct target genes we found T cell differentiation and Th2 pathway as highly enriched pathways.

### Decreased IL-12 cytokine secretion by Zeb1 KD DCs leads to Th2 development

**Figure 8:**
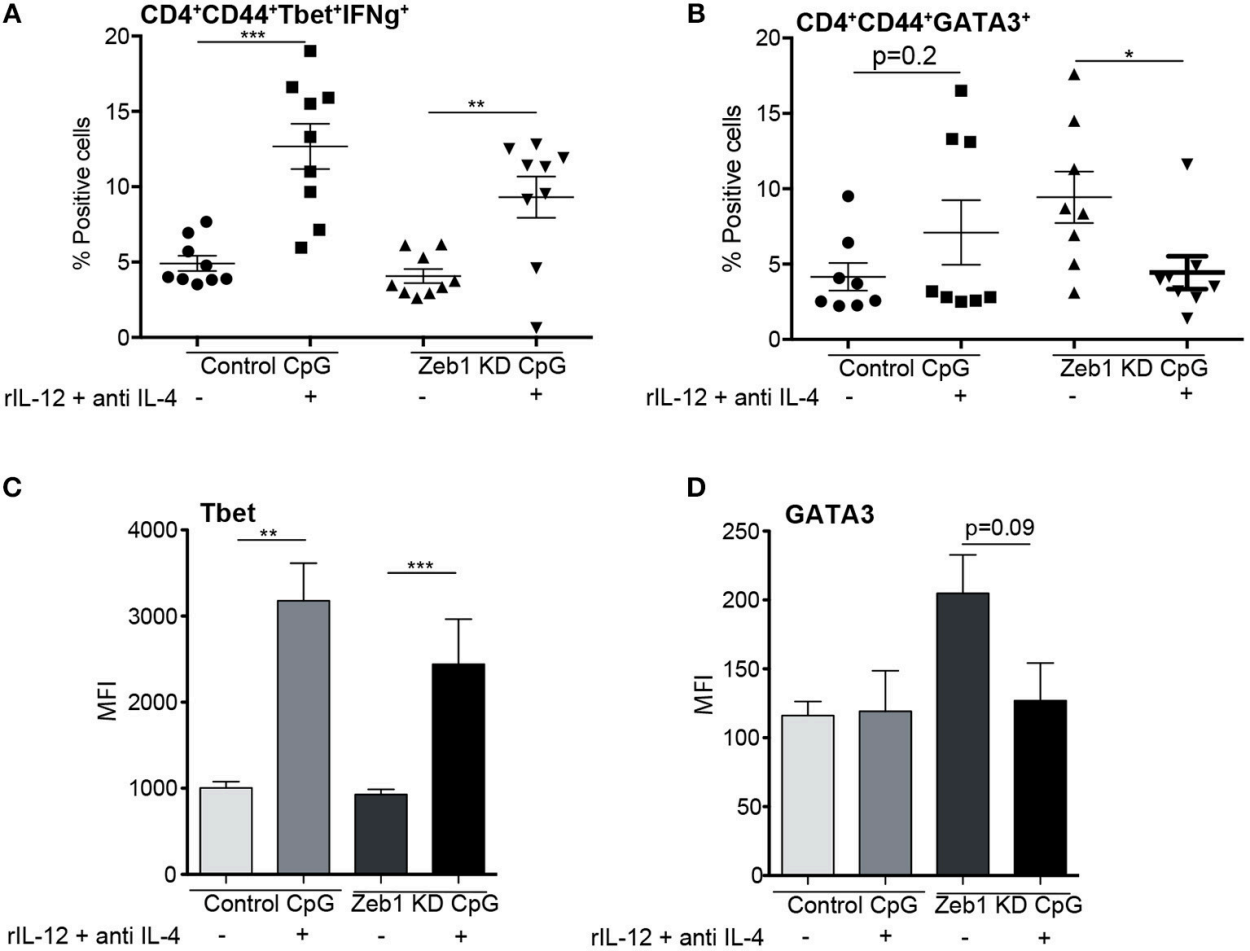
Decreased IL-12 secreted by Zeb1 KD cDC1 DCs is responsible for inducing Th2 differentiation *n* = 8–10. **(A)** Scatter-plot depicting an increase in percentage positive cells for Th1 markers Tbet^+^ IFNγ^+^ in Zeb1 KD DCs upon supplementation of recombinant IL-12 and anti-IL4 cytokine in the culture media during DC -T cell co-culture *n* = 8–10. **(B)** Scatter-plot depicting a significant decrease in percentage positive cells for Th2 marker GATA3^+^ in Zeb1 KD DCs upon addition of recombinant IL-12 cytokine and anti-IL4 in the culture media during DC-T cell co-culture *n* = 8–10. **(C)** Bar-plot representing the MFI for Th1 marker Tbet^+^ in Zeb1 KD DCs upon supplementation of rIL12 and anti-IL4 cytokine in the culture media during DC -T cell co-culture *n* = 8–10. **(D)** Bar-plot representing the MFI for Th2 marker GATA3^+^ in Zeb1 KD DCs upon supplementation of rIL12 and anti-IL4 cytokine in the culture media during DC -T cell co-culture *n* = 8–10. *p*-values are calculated using two tailed unpaired student's *t*-test, error bars represent SEM. * ≤ 0.05, ** ≤ 0.01, *** ≤ 0.001).

Though we found in our genomic analysis that CLRs were significantly down regulated along with IL-10, IL-6, and IL-12p70 cytokines, we were unable to pinpoint specific molecular mediator leading to the induction of Th2 responses by Zeb1 KD DCs. It has been reported extensively that decreased secretion of inflammatory IL-12p70 cytokine by DCs results in default polarization of T cells toward Th2 subtype (22, 28, 31, 69). Therefore we decided to confirm if Zeb1 KD DCs upon supplementation with recombinant IL-12 (rIL-12) containing medium decreased the polarization of Th cells toward Th2 with a concomitant increased Th1 subtype. We found that OT-II Th cells co-cultured with Zeb1 KD DCs supplemented with 5 ng/ml rIL-12 resulted into significantly increased IFNγ expressing Th1 cells comparable to control DCs (Figure 8A and Supplementary Figure 6A). On the contrary there was a significantly decreased expression of GATA3^+^ Th2 cells in Zeb1 KD DCs after rIL-12 supplementation (Figure 8B and Supplementary Figure 6B). The MFI analysis of Tbet, Th1 inducing factor also demonstrated significant increase after rIL-12 addition in Zeb1 KD DCs whereas GATA3 showed insignificant (*p* = 0.09) but decreasing trend (Figures 8C,D). This lead us to conclude that decreased IL-12p70 secreted by activated Zeb1 KD DCs leads to default development of Th2 phenotype. As Il-12p35 is an exclusive subunit of bioactive IL-12 cytokine whereas Il-12p40 subunit is used as a dimerization partner for other cytokines, we suspected that the decreased IL-12 in Zeb1 KD DCs may be due to decreased Il-12p35. Besides, in our RNA-seq data as well we identified that FPKM of IL12p35 gene was ≥2-fold decreased in Zeb1 KD DCs as compared to control cells after 2 h CpG activation (Supplementary Figure 5D). Furthermore, we found IRF4 transcription factor to be significantly increased in Zeb1 KD DCs, which is well reported to repress inflammatory cytokines in DCs to increase Th2 development (Supplementary Figures 6C,D). Moreover, it would be difficult to exclude the effect of other molecules such as CLRs, Stat5b and other cytokines that are differentially regulated in Zeb1 KD DCs and are reported to impact Th cell polarization intoTh2 subtypes.

## Discussion

Dendritic cells (DCs) are one of the major sentinels of the immune system that determines the fate of T cell dependent adaptive responses. Recently it has been proposed that altering the DC responses can be exploited to affect Th cell subtype development and hence the diseases phenotypes (70, 71). In this study, for the first time, we explored the role of EMT factor Zeb1 in DC function and found that Zeb1 depletion has immune-modulatory effects which skews the Th cells toward the Th2 subtype. Besides that, adoptive transfer of Zeb1 KD cDC1 MutuDCs clears helminth infection by inducing IL-13 and IL-5 secreting Th2 cells in MLNs of infected animals. Moreover, we identified that Zeb1 KD suppresses wide-variety of inflammatory response genes including several CLRs. We concluded that decreased secretion of Th1 inducing inflammatory cytokine IL-12 by Zeb1 KD cDC1 resulted into increased Th2 differentiation. Although the impact of other important factors that were differentially expressed in Zeb1 KD DCs cannot be ignored (24).

Zeb1 is a well-known EMT master regulator that induces mesenchymal properties in cancer cells by increasing N-cadherin and Vimentin levels making it more invasive and metastatic. It directly controls the expression of E-cadherin (Cdh1) by binding to its proximal promoter (72–74). We identified that Zeb1 decrease in our CD8a^+^ DCs cDC1 correlated with the levels of Cdh1 and the cells appeared less migratory and smaller in size *in vitro* as compared to control DCs (data not shown). The impact on Cdh1 was so robust that in all our experiments including RNA-seq we used Cdh1 expression as an indicative of Zeb1 levels. It has been reported that decreased activation and co-stimulation of DCs marked by CD80, CD86, MHC-I and MHC-II resulted into suppression of cytokine secretion (21–23, 28, 31). Besides, these moderately activated DCs are either immune-suppressive leading to development of anergic T cells or immune-modulatory resulting into Th2 phenotype, which depends on the extent of DC activation (21, 75, 76). We observed a significantly decreased activation of DCs in Zeb1 KD DCs both before and after CpG activation. This resulted into decreased cytokine levels in our Zeb1 depleted DCs at both transcript and protein levels. Moreover we cannot exclude the weak TCR strength in this context with low CD80, CD86 and MHCII for triggering a Th2 phenotype.

We know that DCs educate naïve Th cells to differentiate into different subtypes (Th1, Th2, Th17 or Tregs) which depends on the state of DC activation and the extracellular milieu containing a cytokine cocktail majorly of IL-4, IL-10, IL-12p70, IL-6, TGFβ along with others (21, 77). The cDC1 DCs are reported to induce strong Th1 effector response as compared to their counterpart cDC2 DCs which have the inherent property of inducing Th2 responses (78). We observed constitutive expression of Zeb1 in DCs, which could be important to maintain the cDC1-mediated induction of Th1 cell differentiation. Th1 subtype is generated if there is higher concentration of inflammatory cytokine IL-12 in the milieu whereas increased IL-10 or TGFβ with IL-6 leads to Tregs or Th17 phenotype respectively (22, 28). Moreover, IL-12p40 is also reported to form dimer in the absence of IL-12p35 subunit, which is a potent inhibitor of IL-12p70 activity (79). In contrast the cytokine IL-4 is considered pertinent for Th2 subtype development but it is not secreted by DCs in general, therefore the mode of Th2 generation is considered as a default subtype in the absence or decreased secretion of IL-12 along with absence of other T cell differentiation modulators (21–23, 28, 30, 31, 77). In addition to cytokine milieu, the cell surface receptors like CLRs such as CLEC7a, CLEC4a, DC-SIGN are also reported to skew speciation of Th subtypes in a pathogen/antigen dependent manner (24, 80, 81). These reports supported the development of the Th2 phenotype by our Zeb1 KD cDC1 MutuDCs as we observed a significant decrease in Th1 and Th17 polarizing CLRs along with inflammatory cytokine IL-12p70 in Zeb1 KD DCs. It has also been reported that DC-SIGN interacts with ICAM-1 on T cells and enhances the activation and proliferation of T cells (82). In addition, the cytokine IL-27 that is reported to suppress Th2 differentiation was significantly decreased in our Zeb1 KD DCs (83). This further adds up as a plausible mechanism of Th2 polarization after Zeb1 depletion.

It has been extensively reported that Th subtype balance i.e., Th1 and Th2 controls the helminth *H. polygyrus* infection in animals. In Balb/C mice that are Th2 prone, the worms are cleared from intestine much faster i.e., 8 weeks as compared to in C57BL/6 animals where the inherent Th1 behavior sustains the worm infection levels for more than 15–20 weeks (66, 68). Therefore we performed adoptive transfer of activated Zeb1 KD cDC1 MutuDCs in C57BL/6 mice to identify if at physiological level Zeb1 KD DCs could skew the Th subtype toward Th2 and thereby leading to increased worm clearance from intestine. We found that treatment of the helminth infected mice with CpG pulsed Zeb1 KD cDC1 MutuDCs cleared the intestinal worms by D31. Even at D10 the egg counts were lower in Zeb1 KD DC treated mice, though we infected all the animals with equal number of larva at D0. We speculate that it was due to adoptive transfer of CpG pulsed Zeb1 KD DCs that secrete suboptimal inflammatory cytokines, at D7 after infection. Furthermore, we also observed that wild type CD8α^+^ cDC1 treated animals showed 4- to 5-fold higher egg counts as compared to PBS treated animals due to their inherent property of inducing Th1 responses *in vivo*. Collectively, this depicted that ZEB1 expression in DCs is pertinent for initial activation of DCs leading to outburst of cytokines important for development of optimal inflammatory Th1 subtypes.

At the mechanistic level it has been demonstrated that Zeb1 acts as a global transcriptional repressor in pre-adipocytes, CD8^+^ T cells and mesenchymal to epithelial transition (MET) process (84). In contrast to its role as transcriptional repressor there are studies indicating Zeb1 as a co-activator, for example, in complex with Yap1 it activates metastatic inducer genes (85). In addition, Gene Set Enrichment Analysis (GSEA) of Zeb1 depleted breast cancer cells showed its activating role in the expression of inflammatory response genes IL-6, IL-8, and IL-1α (37). Though our transcriptome analysis showed Zeb1 as a global repressor with higher number of upregulated genes at all the time points, we observed down-regulation of most of the immunogenic genes including CLRs, IL-6, IL-10, IL-12, and IL-27 in Zeb1 KD DCs as compared to control cells. We also showed here by integrating ChIP-seq and RNA-seq data that Zeb1 does not act as transcriptional repressor for immune response gene cluster as for other pathway genes group. It further substantiates and suggests that Zeb1 forms some differential complex to control the immune response genes or Zeb1 mostly regulates them indirectly at transcript level. Even in the cases of EMT and immune evasion it has been widely reported that EMT process coincides well with the increased inflammatory environment (86–88). It might be possible that Zeb1 upregulation during EMT also results in increased inflammation in the tumor microenvironment.

We would like to conclude with a message that expression of TF Zeb1 is pertinent for CD8α^+^ cDC1 activation leading to immunogenic response generation. It regulates activation and thereby secretion of cytokines by CD8α^+^ cDC1 DCs that are pertinent to induce pathogen/signal specific T cell responses.

## Ethics statement

All the animal experiments were carried following the guidelines of institutional animal ethical committee. The Committee for the Purpose of Control and Supervision of Experiments on Animals (CPCSEA), under the guidelines approved by Institutional Animal Ethics Committee (IAEC).

## Author contributions

SS, AA, BG, HA-O and SR designed the study. SS, AA, VB, MK performed the experiments SS, AA, BG, HA-O and SR did interpretation of the results. AG, SR performed the computational/bio-informatics analysis. SS, HA-O and SR wrote the manuscript. SS, AA, AG, BG, MK, HA-O and SR edited the manuscript.

### Conflict of interest statement

The authors declare that the research was conducted in the absence of any commercial or financial relationships that could be construed as a potential conflict of interest.
