# Supplementary material for: Importance of EMT Factor ZEB1 in cDC1 “MutuDC Line” Mediated Induction of Th1 Immune Response
Source: Front Immunol. 2018 Nov 13;9:2604. doi: 10.3389/fimmu.2018.02604 (PMC6243008; doi:10.3389/fimmu.2018.02604)
Supplement: Supplementary file 9 [file Table_9.DOCX]

**Supplementary figure and table legends**

**Supplementary Figure 1: Immune-profiling of Zeb1 KD and control CD8α^+^ cDC1 MutuDCs upon stimulation with various TLR ligands. n=4-6**

1A. Bar-plot depicting the fold changes in Zeb1 mRNA expression upon CpG stimulation at 2h, 6h and 12h as compared to control DCs. n=5

1B. Bar-plot showing the Zeb1 dCt values normalized to beta actin housekeeping control at various time points in unstimulated (0h) and 2h, 6h and 12h CpG stimulated DCs. n=5

1C. Scatter-plot showing the percentage positive cells for activation and co-stimulation markers CD80, CD86 and MHCII in Zeb1 KD CD8α^+^ cDC1 compared to control cells before and after 12h of pIC and CpG + pIC activation. The corresponding panel depicts bar plots for Median Fluorescence Intensity (MFI) for respective surface markers in Zeb1 KD DCs as compared to control DCs. n=4-6

1D. Scatter-plot showing the percentage positive cells for intracellular cytokines IL-10, IL-12p40, IL-6 in Zeb1 KD cDC1 compared to control cells before and after 12h activation with pIC and CpG + pIC. The corresponding panel depicts bar plot for Median Florescence Intensity (MFI) cytokines in Zeb1 KD DCs with respect to control DCs. n=4-6

1E. Representative histogram plots showing the MFI shifts for surface markers and intracellular cytokine shown in panels 1C and 1D.

1F. Bar-plot depicting the Bio-plex quantitation of GM-CSF, IFNγ, TNF-α cytokines secreted in the supernatant of 12h CpG activated Zeb1 KD and control DCs. n=5

p-values are calculated using two tailed unpaired student’s *t*-test, error bars represent SEM.

* ≤0.05, ** ≤0.01, *** ≤0.001

**Supplementary Figure 2A: *Ex-vivo* immune profiling of Zeb1 depleted DCs derived from bone marrow cultures using FLT3L.** Bar plot depicting MFI plots for surface markers CD86, MHCII, CD40 and PDL1, intracellular cytokine IL10, IL-12p40, IL6 and IL27 in Zeb1 KD and control bone marrow derived DCs. n=6-8

p-values are calculated using two tailed unpaired student’s *t*-test, error bars represent SEM.

* ≤0.05, ** ≤0.01, *** ≤0.001

**Supplementary Figure 2B-2E: Th subtype profiling from DC-T cell co-culture experiment.**

2B. Dot-plots of Th cells co-cultured with Zeb1 KD and control DCs for 96h showing percentage of double positive cells for Tbet^+^ IFNγ^+^  (Th1 subtype) cells in effector CD4^+^CD44^+^ Th cell population. n=8

2C. Scatter-plot of showing percentage positive cells for Tbet^+^ IFNγ^+^ (Th1) cells in effector CD4+CD44+ Th cell population generated in Th cells co-cultured with Zeb1 KD and control DCs for 96h. n=8

2D.Representative bar plots depicting MFI used for showing Th cells subtypes GATA3, IL13 (Th2), Tbet, IFNy (Th1) and FoxP3 (Tregs).

2E. Bar-plot showing the bio-plex quantitation of IL-2 cytokine in the cell culture supernatants of 2h, 6h, 12h and 24h CpG activated Zeb1 KD and control DCs. n=5

2F. Bar-graph showing the dCt values (Ct Zeb1/ Ct β-actin) for Zeb1 transcript expression in different subsets of primary DCs cDC1, cDC2 and pDCs isolated from spleen of FLT3L transgenic C57BL/6 mice. n=3

p-values are calculated using two tailed unpaired student’s *t*-test, error bars represent SEM.

≤0.05, ** ≤0.01, *** ≤0.001

**Supplementary Figure 3: Splenocytes from Balb/C mice co-cultured with CpG activated Zeb1 KD cDC1 enhances their differentiation towards Th2 phenotype. n=4**

3A. Pseudo-colour plot depicting the gating strategy used to identify CD3^+^T cells followed by the CD4^+^ CD44^+^ effector T cells.

3B**.** Scatter-plot from the FACS analysis of Th cells co-cultured with Zeb1 KD and control DCs for 96h showing the percentage positive cells for GATA3^+^, IL4^+^, IL-13^+^, IL5^+^ and IL10^+^ cells in CD4^+^CD44^+^ effector Th cell population. Following panel represents the bar blot for MFI of the same. n=4.

3C. Scatter-plot of Th cells co-cultured with Zeb1 KD and control DCs for 96h showed percentage positive cells and MFI for Tbet^+^ and IFNγ^+^ cells in CD4^+^CD44^+^ effector Th cell population. n=4.

3D. Pseudo-colour plot showing gating strategy followed to demarcate CD8 subtype by identifying CD3^+^ T cells and the CD8^+^ CD44^+^ effector T cells.

3E. Effect on cytotoxic T cell, CTLs (CD8^+^)T cells: Scatter plot showing the percentage positive and MFI for effector CD8^+^CD44^+^ population and granzyme and perforins levels in CD8^+^T cells when Zeb1 KD and control DCs when co-cultured with splenocytes of Balb/C mice. n=4

p-values are calculated using two tailed unpaired student’s *t*-test, error bars represent SEM.

* ≤0.05, ** ≤0.01, *** ≤0.001

**Supplementary Figure 4: Treatment of helminth-infected animals with activated Zeb1 KD DCs enhances worm clearance by increasing Th2 responses (response at D31). n=3**

Scatter-plot showing the detailed immune profiling of CD4^+^CD44^+^ effector Th cells isolated form mesenteric lymph nodes of helminth infected Zeb1 KD treated and control DC treated animals at D31. Percentage positive cells and respective bar-plots showing the MFI shifts for Th2 (IL13 and IL5); Th1 (IFNγ), Tregs (IL10) and cytokine markers. n=3

p-values are calculated using two tailed unpaired student’s *t*-test, error bars represent SEM.

* ≤0.05, ** ≤0.01, *** ≤0.001

**Supplementary Figure 5: Biological pathways enriched for the genes differentially regulated in Zeb1 KD DCs transcriptome analysis as compared to control DCs.**

5A & 5B. Ranked pathways for differentially expressed genes at 0h and 6h CpG activation.

5C. Top enriched pathway for Zeb1 regulated genes found to be annotated at regulated regions bound by Zeb1-PU.1-IRF4 TFs.

5D. Bar plot depicting the mean FPKM showing the expression of IL-12a in RNA-seq data at 0h, 2h, 6h and 12h after CpG activation. Interestingly IL-12a showed drastically increased expression at 2h after CpG activation only. n=2

5E. IGV snap-shot demonstrating the RNA-seq tag-density on IL-12a gene in Zeb1KD and control DCs at 0h, 2h, 6h and 12h after CpG activation. n=2

**Supplementary Figure 6: Decreased IL-12 and elevated IRF4 expression in Zeb1 KD DCs, a possible outcome of Th2 response.**

6A. Representative FACS dot-plot showing the Tbet^+^IFNγ^+^ population in CpG stimulated control and Zeb1 KD cocultured T cells with and without rIL-12 and anti-IL4 supplementation.

6B. Representative FACS dot-plot showing GATA3^+^IL13^+^ population in CpG stimulated control and Zeb1 KD cocultured T cells with and without rIL12 and anti-IL4 supplementation.

6C. Bar-plot depicting the fold change for Irf4 expression in Zeb1 KD compared to control DCs at various time points of CpG stimulation (0h, 2h, 6h and 12h )at mRNA level (qPCR). n=5

6D. Representative western blot analysis for IRF4 protein expression in unstimulated (0h) and 2h, 6h and 12h CpG stimulated Zeb1 KD and control CD8^+^cDC1. Corresponding panel represents densitometric analysis for the same. n=2

**Supplementary table legends**

**Supplementary table 1.** Excel file showing the list of significant (p<0.05, FDR<0.05) differentially expressed genes in RNA-seq of Zeb1 KD and control CD8+ cDC1. Sheet 1: Differentially regulated genes at 0h (without stimulation) of CpG treatment in Zeb1KD and control DCs, Sheet 2: Differential expression of genes post 6h of CpG stimulation, Sheet 3: Differential expression of genes at 12h of CpG pulsed Zeb1 KD and control CD8+cDC1.

**Supplementary table 2.** List of genes in top enriched pathways from 12h post CpG stimulation with FPKM values and log2 fold change in Zeb1 KD CD8^+^cDC1 DCs post 0h, 6h and 12h of CpG treatment. This list has been used to generate heat-map shown in Fig.6c.

**Supplementary table 3.** Excel file having Ingenuity Pathways Analysis (IPA) report showing significantly enriched genes involved with their respective p values at 0h (sheet 1), 6h (Sheet 2) and 12h (Sheet 3).

**Supplementary table 4.** Excel file showing annotated peaks of Zeb1 ChIP-seq data in unstimulated CD8^+n^cDC1 DCs.

**Supplementary table 5.** Excel file showing genes annotated to Zeb1 peaks overlapping with PU.1 and IRF4 binding from publically available ChIP-seq data, to validate the predicted *de novo* motifs analysis. Sheet 1: Excel sheet for the overlapped genes. Sheet 2: Excel sheet for the overlapped pathways.

**Supplementary table 6.** Excel sheet showing the FPKM values for the immune response (GO:0002376) related genes that are regulated by Zeb1. The ChIP-seq binding column depicts whether the gene was found to be bound (1) or unbound (0) by Zeb1. The data from this table was used to generate heat-map represented in Figure 7E.

**Supplementary table 7.** Excel file showing the IPA pathways genes enriched in ChIP-seq and RNA-seq overlap. Ingenuity Canonical Pathways significantly enriched for the genes that were directly bound and regulated by Zeb1 at 0h (Sheet1), after 6h of CpG activation (Sheet2), post 12h of CpG activation (Sheet 3).

**Supplementary table 8.** Resource table enlisting the reagents and materials used in the study (Sheet 1).
