# Supplementary figures and images for: Importance of EMT Factor ZEB1 in cDC1 “MutuDC Line” Mediated Induction of Th1 Immune Response
Source: Front Immunol. 2018 Nov 13;9:2604. doi: 10.3389/fimmu.2018.02604 (PMC6243008; doi:10.3389/fimmu.2018.02604)

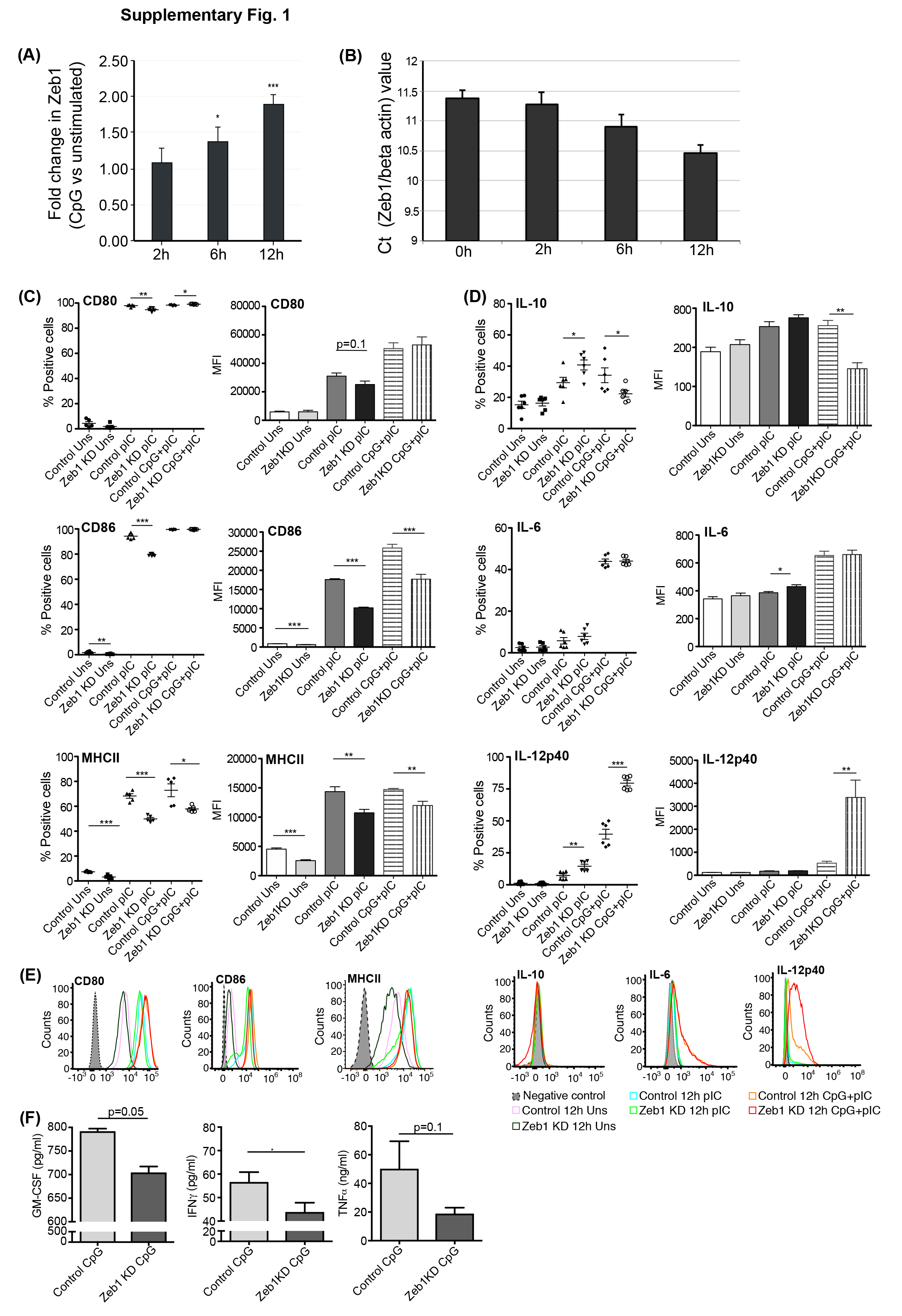

Supplement: Supplementary file 10 [file Image_1.TIF]

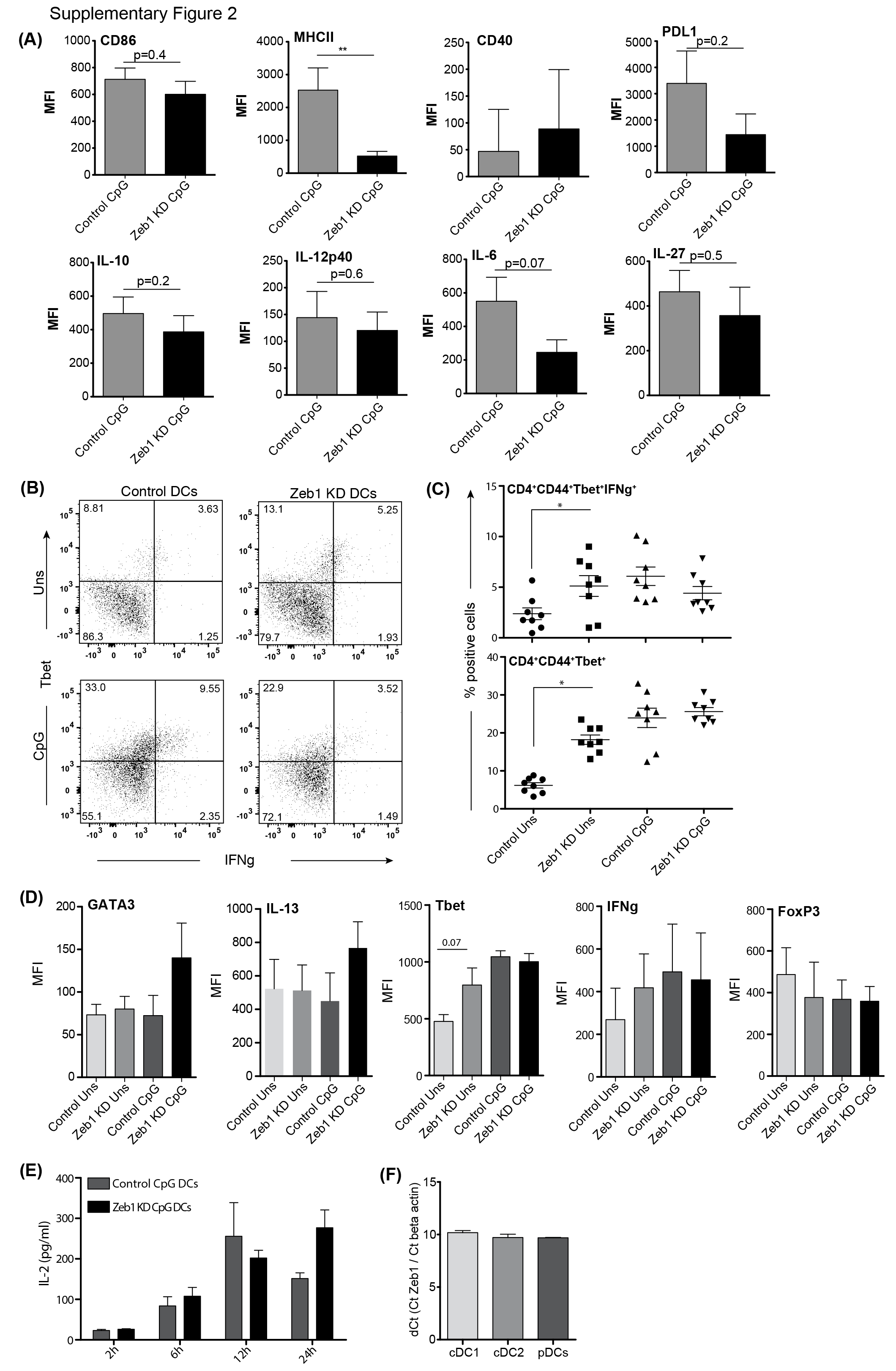

Supplement: Supplementary file 11 [file Image_2.TIF]

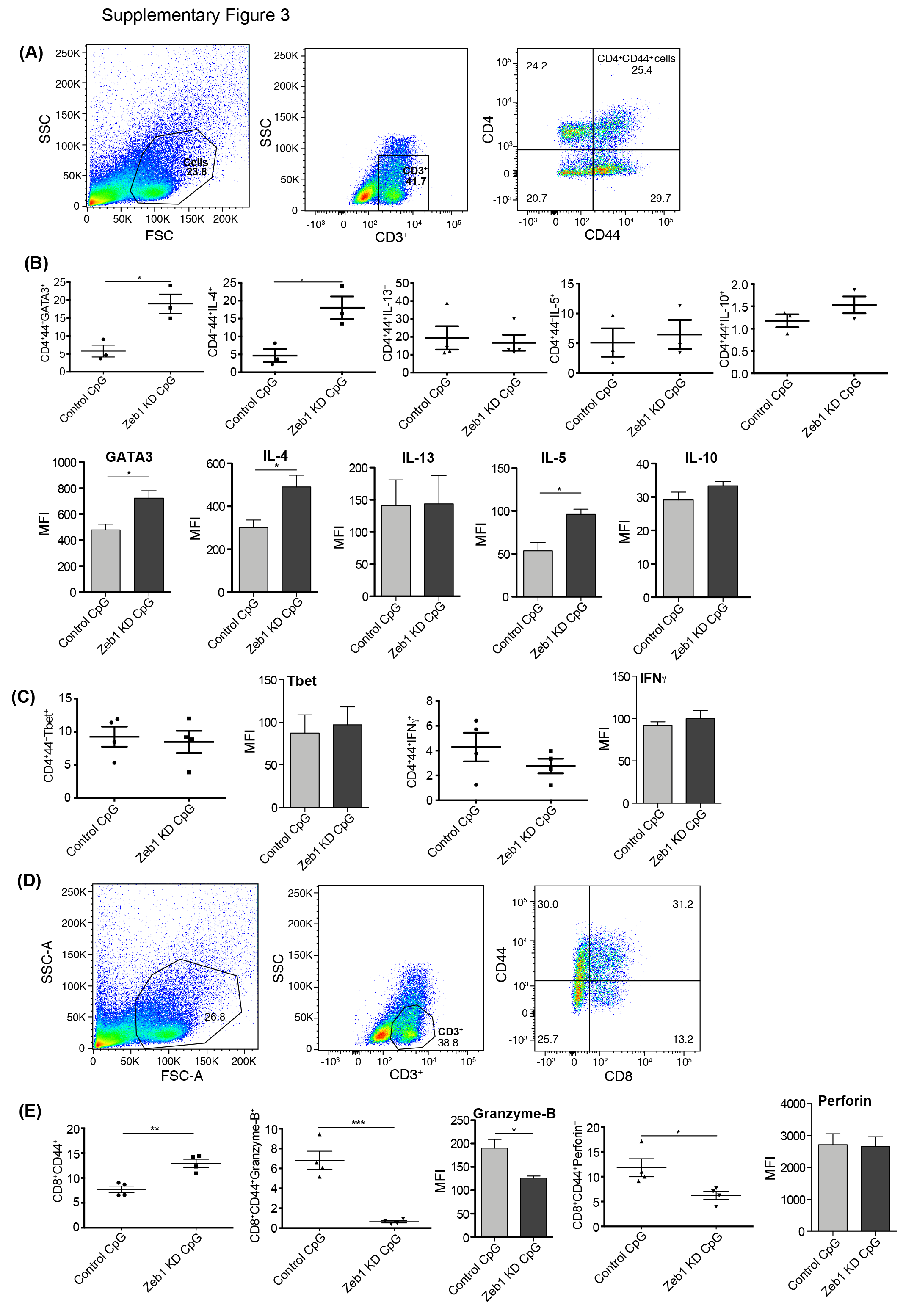

Supplement: Supplementary file 12 [file Image_3.TIF]

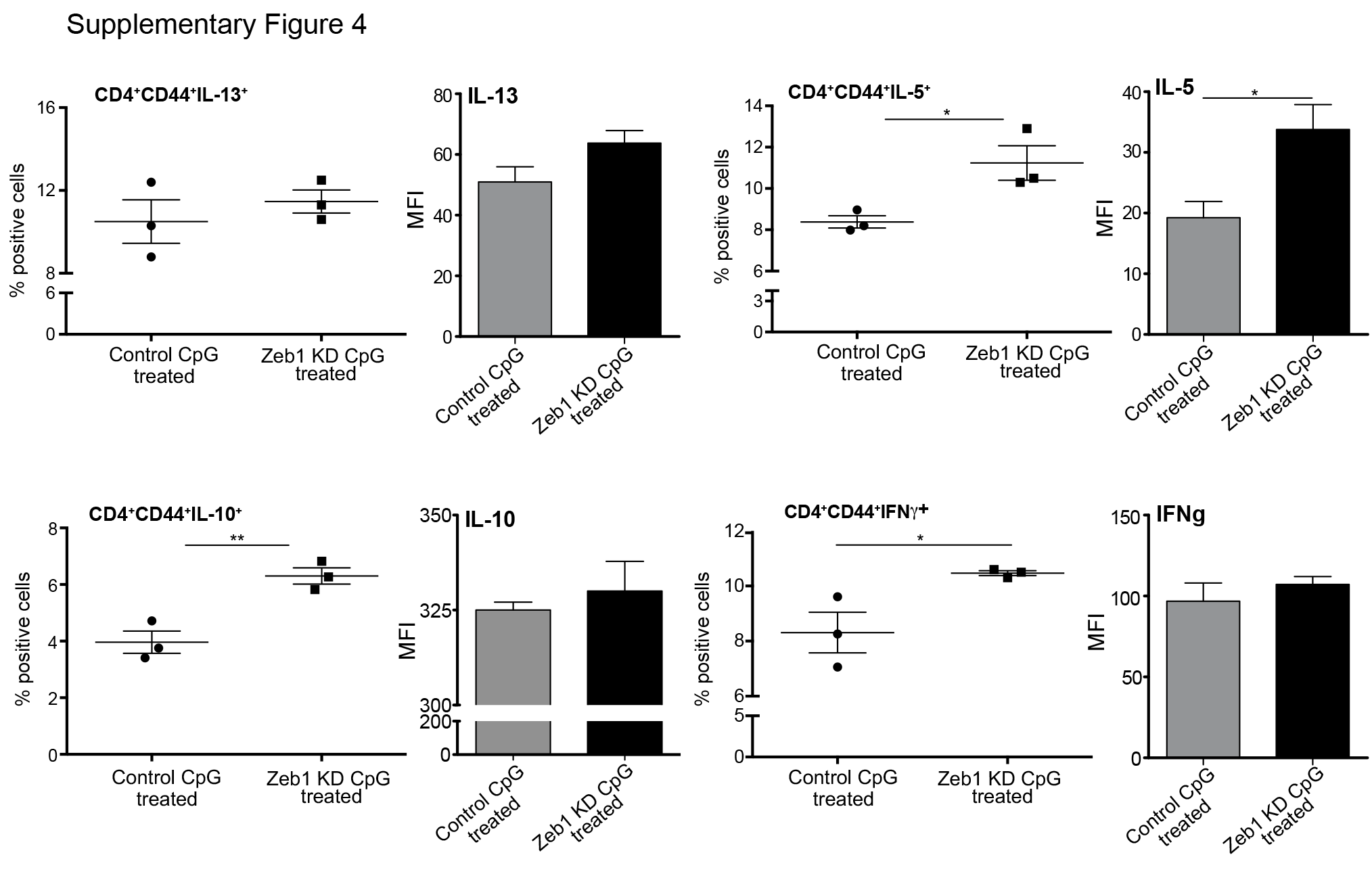

Supplement: Supplementary file 13 [file Image_4.TIF]

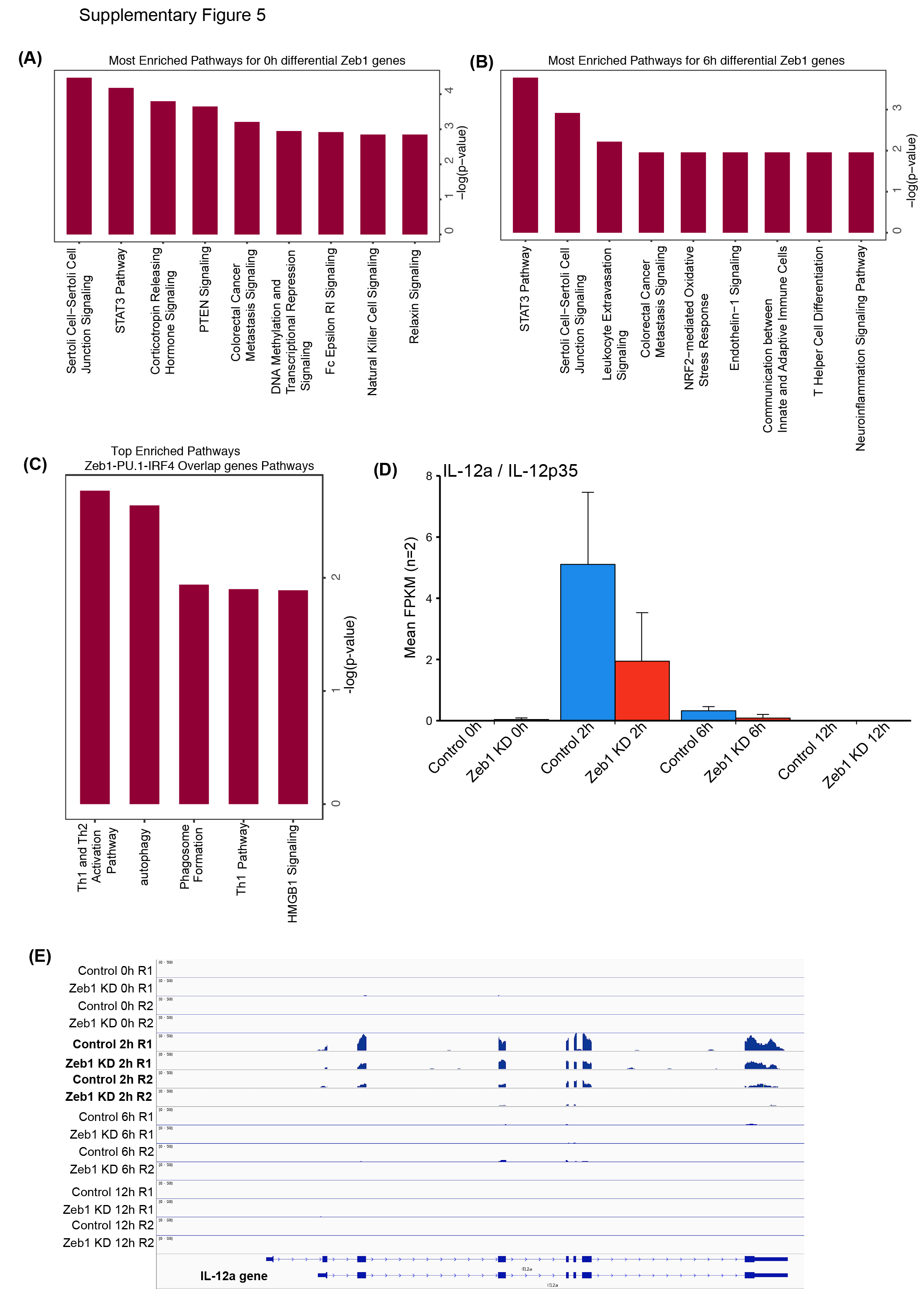

Supplement: Supplementary file 14 [file Image_5.TIF]

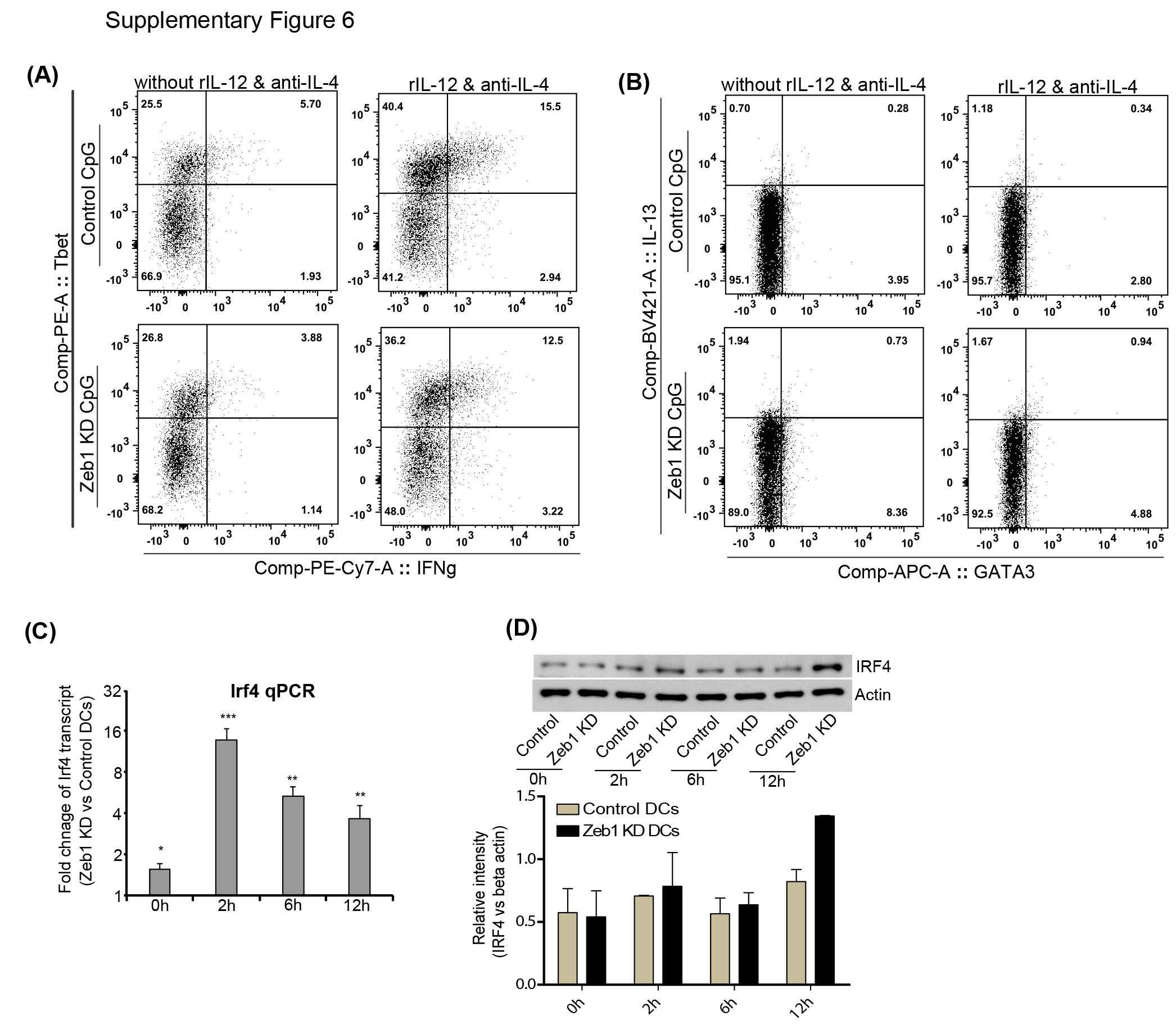

Supplement: Supplementary file 15 [file Image_6.TIF]
